# Supplementary material for: Why urban communities from low-income and middle-income countries participate in public and global health research: protocol for a scoping review
Source: BMJ Open. 2023 Jun 5;13(6):e069340. doi: 10.1136/bmjopen-2022-069340 (PMC10254990; doi:10.1136/bmjopen-2022-069340)
Supplement: Author's manuscript [file bmjopen-2022-069340.draft_revisions.pdf]

BMJ Open is committed to open peer review. As part of this commitment we make the peer review history of every article we publish publicly available.

When an article is published we post the peer reviewers' comments and the authors' responses online. We also post the versions of the paper that were used during peer review. These are the versions that the peer review comments apply to.

The versions of the paper that follow are the versions that were submitted during the peer review process. They are not the versions of record or the final published versions. They should not be cited or distributed as the published version of this manuscript.

BMJ Open is an open access journal and the full, final, typeset and author-corrected version of record of the manuscript is available on our site with no access controls, subscription charges or pay-per-view fees (http://bmjopen.bmj.com).

If you have any questions on BMJ Open's open peer review process please email info.bmjopen@bmj.com

BMJ Open

Why urban communities from low- and middle-income countries participate in public and global health research: Protocol for a scoping review

Journal:	*BMJ Open*
Manuscript ID	bmjopen-2022-069340
Article Type:	Protocol
Date Submitted by the Author:	19-Oct-2022
Complete List of Authors:	Gagnon-Dufresne, Marie-Catherine; University of Montreal, School of Public Health<br>Sarmiento, Ivan; McGill University, Family Medicine; Universidad Del Rosario, Escuela de Medicina y Ciencias de la Salud<br>Fortin, Geneviève; University of Montreal, School of Public Health<br>Andersson, Neil; McGill University,<br>Zinszer, Kate; Universite de Montreal Institut de recherche en sante publique, Department of Social and Preventive Medicine
Keywords:	PUBLIC HEALTH, PREVENTIVE MEDICINE, QUALITATIVE RESEARCH

SCHOLARONE™
Manuscripts

Why urban communities from low- and middle-income countries participate in public and global health research: Protocol for a scoping review

CORRESPONDING AUTHOR

Marie-Catherine Gagnon-Dufresne
École de santé publique, Université de Montréal
7101 Park Avenue, Montréal, QC
Canada H3N 1X9
marie-catherine.gagnon-dufresne@umontreal.ca

AUTHORS

Marie-Catherine Gagnon-Dufresne[1,2,3*], Iván Sarmiento[3,4], Geneviève Fortin[1,2], Neil Andersson[3,5], Kate Zinszer[1,2]

1. École de santé publique, Université de Montréal, Montréal (Canada)
2. Centre de recherche en santé publique (CReSP), Montréal (Canada)
3. CIET-PRAM, Department of Family Medicine, McGill University, Montréal (Canada)
4. Grupo de Estudios en Sistemas Tradicionales de Salud, Escuela de Medicina y Ciencias de la Salud, Universidad del Rosario, Bogotá (Colombia)
5. Centro de Investigación de Enfermedades Tropicales (CIET), Universidad Autónoma de Guerrero, Acapulco (Mexico)

WORD COUNT

3,362 words

For peer review only

Why urban communities from low- and middle-income countries participate in public and global health research: Protocol for a scoping review

ABSTRACT

Introduction: As the number of people living in cities increases worldwide, particularly in low- and middle-income countries (LMICs), urban health is a growing priority of public and global health. Rapid and unplanned urbanization in LMICs has increased health inequalities, putting the urban poor at increasing risk of ill health due to difficult living conditions in cities. Collaboration with underserved urban communities in research is a key strategy for addressing the diverse challenges they face. The objective of this scoping review is therefore to identify factors that influence the participation of urban communities from LMICs in public and global health research.

Methods and analysis: With the help of a health librarian, we will develop a search strategy to explore the following databases: MEDLINE, Embase, Web of Science, Cochrane, Global Health, CINAHL, and Google Scholar. We will use MeSH terms and keywords to look at empirical research conducted in French or in English. There will be no restriction in terms of dates of publication. Two independent reviewers will screen and select studies, first based on titles and abstracts, and then on full text. Two reviewers will extract data. We will summarize the results using tables and fuzzy cognitive mapping.

Ethics and dissemination: This scoping review is part of a larger project to be approved by the University of Montréal's Research Ethics Committee for Science and Health in Montréal (Canada), and the Institutional Review Board of the James P. Grant School of Public Health at BRAC University in Dhaka (Bangladesh). Results from the review will contribute to a participatory process seeking to combine scientific evidence with the experiential knowledge of stakeholders in Dhaka to understand how to better collaborate with communities for research. The review could contribute to a shift towards research that is more inclusive and more beneficial for communities.

KEYWORDS

public health, global health, community-based participatory research, stakeholder participation, urban population, urban health, fuzzy logic

STRENGTHS AND LIMITATIONS OF THIS STUDY

- The review will systematize the wide array of factors that influence the participation of urban communities from low-and middle-income countries in public and global health research.

For peer review only

- Reporting on community participation is heterogeneous, and identifying the research approaches, health issues, contexts, and community characteristics that favor participation will be challenging.
- The scoping review will summarize results using fuzzy cognitive mapping, providing a compatible format for contextualizing the literature in the views of local stakeholders.

INTRODUCTION

As the number of people living in cities increases worldwide, particularly in low- and middle-income countries (LMICs), the health of urban populations is a growing priority of public and global health.[1] Urbanization brings changes to the disease burdens, determinants of health, and patterns of health inequalities.[1,2] Despite the benefits of urban living and progress in population health, rapid and unplanned urbanization in LMICs has worsened health inequalities.[3] Not everyone in cities experiences these improvements equally, as policies and other efforts often fail to reach the most marginalized communities, including those living in informal settlements.[3] The urban poor are therefore at increased risk of ill health due to the difficult living conditions in cities.[3,4]

Several researchers in public and global health have criticized the reproduction of colonial relations in efforts to improve the health of populations in LMICs, as these efforts are often led by foreign researchers with little input from local populations.[5–7] The resulting unequal power dynamics between researchers and communities are among the reasons research makes little or mixed contributions to health.[8] In response, there are increasing calls to decolonize public and global health through community participation in research, to better meet their needs and ensure local relevance of the initiatives put in place to improve their health.[8–10]

Community participation in health research is recognized for building capacity and fostering conditions to enable better community control over determinants of their health.[11,12] Community participation can lead to equitable partnerships between communities and researchers, making research more empowering and effective.[12] However, most health research uses top-down community engagement approaches rather than bottom-up participatory methods.[13] While there is no standard definition of community participation, different uses of the term form a continuum from consulting or informing communities to sharing power with them.[13,14]

Collaborating with marginalized urban communities is a key strategy for addressing the many challenges they face.[3,15,16] Yet, these communities represent a particularly hard-to-reach group, as asymmetries in access to resources and opportunities affect their capacity to fully participate in and benefit from research implement to improve their health.[15,17] There is therefore an urgent need to better understand the barriers and enablers to their participation.[18–20]

For peer review only - http://bmjopen.bmj.com/site/about/guidelines.xhtml

The objective of this scoping review is to identify factors that influence the participation of urban communities from low- and middle-income countries in public and global health research. Part of a larger project, this scoping review will contribute to a dialogue between scientific and experiential knowledge on the factors that influence community participation in public and global health research. We will contextualize the results from the scoping review in the views of stakeholders in Dhaka (Bangladesh) in a participatory process to reflect their experiences. This contextualization will identify barriers and enablers to participation that are specific to Dhaka, in preparation for a cluster randomized controlled trial testing the effect of a participatory community mobilization intervention for reducing dengue infection.[21]

METHODS AND ANALYSIS

This review is part of a larger project aimed at comparing and combining different knowledge sources to provide a knowledge base for decision making, that will be used to inform a cluster randomized controlled trial to reduce dengue infection in Dhaka.[21] The larger project will consider four knowledge sources: 1) the scoping review described in this protocol; 2) the views of Canadian and Bangladeshi public and global health researchers; 3) the views of personnel from community-based organizations in Dhaka; 4) the views of community members from underserved neighbourhoods in Dhaka. We will adapt the *Weight of Evidence* approach [22,23] and use fuzzy cognitive mapping to bring these different knowledge sources into conversation.

In this protocol, we focus on describing the procedures to conduct the scoping review and briefly discuss how the results will be used to inform the subsequent phases of the larger project. The proposed scoping review will be conducted in accordance with the PRISMA-ScR (Preferred Reporting Items for Systematic Reviews and Meta-Analyses – Extension for Scoping Reviews) guidelines and Joanna Briggs Institute's methodology to ensure accuracy, completeness and transparency.[24–27]

Review questions

This scoping review will seek to answer the following question: What factors influence the participation of urban communities from low- and middle-income countries in research, based on evidence from the public and global health literature?

The review will also seek to answer the following sub-questions:

1) What are the main barriers and enablers of community participation in public and global health research?
2) What is the relative influence of these factors on community participation?
3) What research approaches are most and least favourable to community participation?
4) What public and global health issues are most and least favourable to community participation?
5) What contexts are most and least favourable to community participation?
6) What community characteristics are most and least favourable to community participation?

For peer review only - http://bmjopen.bmj.com/site/about/guidelines.xhtml

Eligibility criteria

Types of sources

This scoping review will consider empirical studies with quantitative, qualitative, and mixed methods designs. We will not include literature reviews and meta-analyses, but we will consider including the empirical studies reported in reviews and meta-analyses if relevant. We will not consider literature reporting on programs, policy or other initiative implemented outside of research purposes.

Participants

This review will look at research participation at the community level rather than at the individual level, therefore excluding studies discussing the participation of individuals in research (*i.e.*, patient engagement, individual motivation to participate). Communities refer to population groups and the locus (*i.e.*, place, venue, or other units) of their actions.[28] Communities can be understood as groups of people with diverse characteristics that are linked by social ties or identities; share common interests or concerns; and engage in joint action in settings, venues or areas that may be physically, geographically, culturally or politically defined.[28–30] The definition of what constitutes a community remains broad for this scoping review but will focus on urban communities in low- and middle-income countries. This is justified by the fact that the larger project is part of a cluster randomized controlled trial on dengue which will be conducted in Dhaka (Bangladesh), and that the factors influencing community participation in research will likely vary between rural and urban communities, and between high- and low- and middle-income countries.

Concept

Community-engaged research is a broad topic, defined in various ways and used for various reasons. It is often an umbrella term for research involving the participation of non-academic stakeholders, with diverse models and conceptual frameworks.[14] There is no standard definition of community participation in research in public and global health.[13,14] The distinction between 'engagement,' 'mobilization' and 'participation' in research is unclear, as these terms are often used interchangeably and with changing definitions.

Various authors discuss the levels of community participation in research as being positioned along a continuum, ranging from information provision and exchange, to consultation, to co-production, and to shared leadership and community control.[14,31–34] For this scoping review, we will not restrict the search to a specific level of participation, but we will examine and compare how different approaches (i.e., community mobilization interventions, partnered research, community-based participatory research designs, etc.) are found to enable or hinder participation. However, research in which there is little community involvement (*i.e.*, health education, consultation efforts in which communities have no decision-making power over some aspects of the research) will be excluded.

For peer review only

Context

This review will focus on communities located in urban settings in low- and middle-income countries. The definition of low- and middle-income countries used for the review will use the World Bank's classification from the 2023 fiscal year, based on countries' gross national incomes per capita.[35] The Cochrane Effective Practice and Organisation of Care (EPOC) group has developed a filter for literature reviews based on the World Bank classification to identify studies relevant to LMICs.[36]

There is no standard international definition of what constitutes an urban setting. Each country has its own definition, following nationally defined criteria on population size, population density, type of economic activity, physical characteristics, level of infrastructure, or other characteristics.[37] Considering the lack of a common definition, the scoping review will consider all studies in LMICs conducted in urban settings or cities as identified by the authors, including neighbourhoods and informal settlements (slums) in cities.

Exclusion criteria

To ensure the selection of relevant studies for the review, we will use the following exclusion criteria:

- Not empirical research
- Discussing community engagement, participation, partnership, or mobilization in contexts other than research (*i.e.*, programs, policy, urban planning)
- Focused on individual engagement in research (*i.e.*, patient engagement, individual motivation)
- Not discussing factors that influence community participation in research
- Conducted in contexts other than urban settings
- Conducted in countries other than low- and middle-income countries
- Full text of the reference is not available

Search strategy

The search strategy will be developed with the help of a health librarian. It will explore the following databases: MEDLINE, Embase, Web of Science, Cochrane, Global Health, CINAHL, and Google Scholar. We will use MeSH terms and keywords to identify studies reported in English or French. We will not have restrictions in terms of dates of publication. We will not contact the authors of the articles selected to request additional information. Table 1 presents the initial search strategy for MEDLINE, which will be adapted for each database.

Table 1. Search strategy for MEDLINE

**Concept 1: Low- and middle-income countries**
1. (afghanistan or albania or algeria or american samoa or angola or "antigua and barbuda" or antigua or barbuda or argentina or armenia or armenian or aruba or azerbaijan or bahrain or bangladesh or barbados or "republic of Belarus" or belarus or byelarus or belorussia or byelorussian or belize or british honduras or benin or dahomey or bhutan or bolivia or "bosnia and herzegovina" or bosnia or herzegovina or botswana or bechuanaland or brazil or brasil or bulgaria or burkina faso or burkina fasso or upper volta or burundi or urundi or cabo verde or cape verde or cambodia or kampuchea or khmer republic or

For peer review only

cameroon or cameron or cameroun or central african republic or ubangi shari or chad or chile or china or colombia or comoros or comoro islands or iles comores or mayotte or "democratic republic of the congo" or democratic republic congo or congo or zaire or costa rica or "cote d’ivoire" or "cote d’ ivoire" or cote divoire or cote d ivoire or ivory coast or croatia or cuba or cyprus or czech republic or czechoslovakia or djibouti or french somaliland or dominica or dominican republic or ecuador or egypt or united arab republic or el salvador or equatorial guinea or spanish guinea or eritrea or estonia or eswatini or swaziland or ethiopia or fiji or gabon or gabonese republic or gambia or "georgia (republic)" or georgian or ghana or gold coast or gibraltar or greece or grenada or guam or guatemala or guinea or guinea bissau or guyana or british guiana or haiti or hispaniola or honduras or hungary or india or indonesia or timor or iran or iraq or "isle of man" or jamaica or jordan or kazakhstan or kazakh or kenya or "democratic people’s republic of korea" or "republic of korea" or north korea or south korea or korea or kosovo or kyrgyzstan or kirghizia or kirgizstan or kyrgyz republic or kirghiz or laos or lao pdr or "lao people's democratic republic" or latvia or lebanon or lebanese republic or lesotho or basutoland or liberia or libya or libyan arab jamahiriya or lithuania or macau or macao or "republic of north macedonia" or macedonia or madagascar or malagasy republic or malawi or nyasaland or malaysia or malay federation or malaya federation or maldives or indian ocean islands or indian ocean or mali or malta or micronesia or "federated states of Micronesia" or kiribati or marshall islands or nauru or northern mariana islands or palau or tuvalu or mauritania or mauritius or mexico or moldova or moldovian or mongolia or montenegro or morocco or ifni or mozambique or portuguese east africa or myanmar or burma or namibia or nepal or netherlands antilles or nicaragua or niger or nigeria or oman or muscat or pakistan or panama or papua new guinea or new guinea or paraguay or peru or philippines or philipines or phillipines or phillippines or poland or "polish people's republic" or portugal or portuguese republic or puerto rico or romania or russia or russian federation or ussr or soviet union or "union of soviet socialist republics" or rwanda or ruanda or samoa or pacific islands or polynesia or samoan islands or navigator island or navigator islands or "sao tome and principe" or saudi arabia or senegal or serbia or seychelles or sierra leone or slovakia or slovak republic or slovenia or melanesia or solomon island or solomon islands or norfolk island or norfolk islands or somalia or south africa or south sudan or sri lanka or ceylon or "saint kitts and nevis" or "st. kitts and nevis" or saint lucia or "st. lucia" or "saint vincent and the grenadines" or saint vincent or "st. vincent" or grenadines or sudan or suriname or surinam or dutch guiana or netherlands guiana or syria or syrian arab republic or tajikistan or tadjikistan or tadzhikistan or tadzhik or tanzania or tanganyika or thailand or siam or timor leste or east timor or togo or togolese republic or tonga or "trinidad and tobago" or trinidad or tobago or tunisia or turkey or turkmenistan or turkmen or uganda or ukraine or uruguay or uzbekistan or uzbek or vanuatu or new hebrides or venezuela or vietnam or viet nam or middle east or west bank or gaza or palestine or yemen or yugoslavia or zambia or zimbabwe or northern rhodesia or global south or "africa south of the sahara" or sub-saharan africa or subsaharan africa or africa, central or central africa or africa, northern or north africa or northern africa or magreb or maghrib or sahara or africa, southern or southern africa or africa, eastern or east africa or eastern africa or africa, western or west africa or western africa or west indies or indian ocean islands or caribbean or central america or latin america or "south and central america" or south america or asia, central or central asia or asia, northern or north asia or northern asia or asia, southeastern or southeastern asia or south eastern asia or southeast asia or south east asia or asia, western or western asia or europe, eastern or east europe or eastern europe or developing country or developing countries or developing nation? or developing population? or developing world or less developed countr* or less developed nation? or less developed population? or less developed world or lesser developed countr* or lesser developed nation? or lesser developed population? or lesser developed world or under developed countr* or under developed nation? or under developed population? or under developed world or underdeveloped countr* or underdeveloped nation? or underdeveloped population? or underdeveloped world or middle income countr* or middle income nation? or middle income population? or low income countr* or low income nation? or low income population? or lower income countr* or lower income nation? or lower income population? or underserved countr* or underserved nation? or underserved population? or underserved world or under served countr* or under served nation? or under served population? or under served world or deprived countr* or deprived nation? or deprived population? or deprived world or poor countr* or poor nation? or poor population? or poor world or poorer countr* or poorer nation? or poorer population? or poorer world or developing econom* or less developed econom* or lesser developed econom* or under developed econom* or underdeveloped econom* or middle income econom* or low income econom* or lower income econom* or low gdp or low gnp or low gross domestic or low gross national or lower gdp or lower

gnp or lower gross domestic or lower gross national or lmic or lmics or third world or lami countr* or transitional countr* or emerging economies or emerging nation?).ti,ab,sh,kf.<br>2. Developing countries/<br>3. 1 or 2
**Concept 2: Community participation in research**
4. (((participat* or communit* or partner*) adj3 research) or (communit* adj3 (participat* or engage* or mobili?ation or intervention*)) or participatory or CBPR).ti,ab,sh,kf.<br>5. Community-based participatory research/<br>6. Community participation/<br>7. 4 or 5 or 6
**Concept 3: Urban settings**
8. (urban* or city or cities or metropol* or megacit* or megalop* or municipalit* or "informal settlement" or "informal settlements" or slum* or favela* or "shanty town" or "shanty towns" or ghetto* or bustee*).ti,ab,sh,kf.<br>9. Urban Health/<br>10. Urban Population/<br>11. Cities/<br>12. Urbanization/<br>13. Poverty Areas/<br>14. 8 or 9 or 10 or 11 or 12 or 13
**Final search strategy**
15. 3 and 7 and 14

The search strategy will be developed with the input from a librarian and the research team to identify new keywords.[38] After our initial screening in MEDLINE, we will search the included articles for new keywords. A new search will then be conducted combining the newly found MeSH terms and keywords to the existing search. A librarian will assess whether these new terms should be included in the final search strategy. When all articles are screened, we will search the reference lists of selected studies to identify additional studies meeting our inclusion criteria.

Study selection

Following the search, all identified citations will be collated and uploaded into Covidence,[39] and duplicates will be removed. Study selection will be conducted in two phases by two independent reviewers, who will reconcile differences by consensus. A third independent reviewer will help resolve any further disagreement.

The initial screening of the retrieved sources will use titles and abstracts. The second phase of selection will use full text. Reasons for excluding sources at full text that do not meet the inclusion criteria will be recorded and reported in the review. The results of the search and the study selection process will be reported in the flow diagram developed by PRISMA-ScR.[27,40]

Data extraction

Two reviewers will develop and pilot a data extraction form, and extract the data in Covidence.[39] The form will include:

a. Details on the study (title, names of the authors, year of publication, study objectives, research design, and data collection and analysis methods)

For peer review only

b. The country and the urban contexts in which studies were conducted
c. The communities targeted
d. The participation approach used and the extent of community participation
e. The findings regarding the factors (barriers, enablers, and other factors) influencing the participation of urban communities in public and global health research
f. If available, the relative influence (qualitative or quantitative) of the factors identified on community participation
g. If available, other relations among the factors identified, and their relative influence on community participation
h. Explanation of the relationships between factors (quotes from the articles)

We will not systematically extract data on the results of the studies since this is outside the scope of the review objectives and research question.

The data extraction form will be piloted before beginning the study selection process with a random sample of 5 studies among all the studies to be reviewed. The pilot test will help identify missing data and will contribute to ensuring that the reporting of participation approaches and factors influencing community participation is coherent across studies and between the two reviewers. The data extraction form will be modified and revised as necessary, in an iterative manner, during the data extraction process. Modifications will be detailed in the report of the review.

Any disagreements on data extraction that arise between the two reviewers at the pilot or data extraction stages will be resolved through by consensus, or by discussion with a third independent reviewer if necessary.

Data analysis and presentation

The presentation of results will follow the PRISMA-ScR guidelines.[27] We will present the results in tables and use fuzzy cognitive mapping (FCM) to illustrate how the different factors identified influence community participation in research, adapting the *Weight of Evidence* approach.[22,41] A narrative summary will also accompany the tabulated and mapped results, describing how the results relate to the review objectives and questions.

FCM uses graph theory and fuzzy logic to generate soft models of how change could happen based on assumed causal relationships.[42–44] These soft models are illustrated through graphs called fuzzy cognitive maps (Figure 1), which are used to represent assumed causal relationships between concepts.[45,42] The maps use nodes (factors affecting the issue) and edges (arrows representing the relationships between factors), weighted by the relative strength of their influence on the issue of interest.[46,42,22] Depending on the knowledge source of the maps, edges can have different values (hence the term *fuzzy*) to quantify their influence in a relative way.[44]

FCM will be the cornerstone for the presentation of the scoping review, through the creation of fuzzy cognitive maps to represent: 1) each article included in the review; and 2) a composite map for the whole review. FCM will allow to summarize in a composite map the relative influence that each factor might have on community participation, in

For peer review only

relation to all the other factors identified in the review.[22,23,42] We will go through several steps (detailed below) to create the composite literature-based fuzzy cognitive map of the barriers and enablers to community participation (Figure 2).

First, we will create one fuzzy cognitive map for each article selected in the scoping review. In each individual map, community participation will be the outcome of interest. We will include each barrier and enabler of community participation mentioned in the article (point e in the data extraction form) as a node in the map, which we will organize in a table. This table will have two initial columns indicating the origin factor (from) and the consequence factor (to). Additional columns will present the evidence supporting the relationship between both factors from the article (point h in the data extraction form). Each relationship identified will be a row in the table.[44]

Second, once all the individual tables are created, we will standardize the names of the factors across the individual articles so that they can be comparable.[41] On each individual map reporting the relationships identified in each study, we will calculate fuzzy transitive closure in the open access CIETmap 2.0.[47] Fuzzy transitive closure is a mathematical model used to calculate the influence of each relationship on community participation, considering all the possible relationships represented in the map.[48,49] After transitive closure, each relationship will have a value between 0 (having no influence) and 1 (having the strongest influence) to represent the relative strength of their influence on community participation, with positive and negative signs indicating whether the relationship is stimulative or inhibitive.[22,48]

Third, we will create a composite map for the whole review. To create this composite map, we will attribute weights to each node using Harris' discourse analysis, an analytical approach developed in the 1950s based the frequency of occurrence of discourse elements sharing similar meanings in a body of text, such as a literature review.[50,41] We will consider the frequency of occurrence of each relationship across the maps developed for each article in the scoping review. This means that a factor that is repeated in multiple maps would have a stronger causal meaning for community participation than a factor only mentioned in one or two maps.[41] We will establish the relative frequency of factors by dividing each occurrence by the highest frequency across all the maps. We will therefore obtain a value between 0 for the relationships that did not exist and 1 for the relationship most frequently mentioned.[41] These different steps will allow us to create a composite map representing all the factors identified in the scoping review, weighted according to their relative frequency.

Patient and public involvement

We will include a consultation phase in the scoping review, as Arksey & O'Malley (2005) recognize the benefit of discussing the results of a review with experts.[51] The *Weight of Evidence* approach, which we will adapt for this scoping review, advocates for experiential knowledge to be considered on an equal footing with the evidence synthesized from the literature.[22] Therefore, in the context of the larger project, which adopts a participatory methodology and involves a community advisory board, people concerned with the issue

For peer review only - http://bmjopen.bmj.com/site/about/guidelines.xhtml

of interest (*i.e.*, participation of urban communities in health research) will be invited to contextualize the scoping review.

After conducting the scoping review, stakeholders in Dhaka will develop their own fuzzy cognitive maps on the factors that they believe can influence community participation in the Bangladeshi context. After developing their maps, they will interpret the literature-based map from the scoping review by comparing the results with their own maps. We will seek the perspectives of three stakeholder groups, namely public and global health researchers, community-based organizations, and community stakeholders.

Finally, we will use the composite map from the scoping review and the various maps from these three stakeholder groups to generate a final map incorporating these two knowledge sources. The literature-based map, the stakeholder maps and this final map will be reviewed through deliberative dialogue with stakeholders in Dhaka.[52] The maps and discussions with stakeholders will inform decision-making for the cluster randomized controlled trial on dengue testing a participatory community mobilization intervention, where communities in Dhaka will develop their own solutions to reduce dengue infection. These steps will be conducted and reported separately.

ETHICS AND DISSEMINATION

This scoping review does not require ethics approval. However, the consultation process is part of a larger project which will need to be approved by the University of Montréal's Research Ethics Committee for Science and Health in Montréal (Canada), and the Institutional Review Board of the James P. Grant School of Public Health at BRAC University in Dhaka (Bangladesh). We will share the results from the scoping review with the scientific community through scientific articles and presentations at conferences, and with local stakeholders in Dhaka through a participatory process involving fuzzy cognitive mapping and deliberative dialogue. Results from this process will directly inform the implementation of the cluster randomized controlled trial on dengue in Dhaka.[21]

CONCLUSION

This protocol described a scoping review which will seek to identify and map the factors that can influence the participation of urban communities from low- and middle-income countries in public and global health research. The review will contribute to the understanding of how to foster the participation of these communities in research, so that it can better respond to local needs. Given that marginalized urban communities represent a particularly hard-to-reach group in research and that urban health is a growing priority of public and global health, findings from this review will be useful for researchers and communities who wish to collaborate to improve population health.

The use of the *Weight of Evidence*, an innovative approach to knowledge synthesis whereby scientific and experiential knowledge are brought into conversation, will allow for the contextualization of the scoping review in the lived experience of stakeholders in Dhaka.[22,41,53] The use of fuzzy cognitive mapping to synthesize the results from the

For peer review only

scoping review offers an operator-independent way to analyze and communicate the relative influence of the factors identified on community participation. The literature-based map will in turn inform a mapping process involving stakeholders from Dhaka (Bangladesh), as part of the larger project. This scoping review offers an example of the contextualization of scientific evidence in the views of stakeholders through the *Weight of Evidence* [22,23,53], which opens the possibility for contextualizing other literature reviews in lived experience anywhere.

Better understanding the factors that influence the participation of communities in research could support a shift from researcher-driven health research towards research that is more inclusive of community voices and needs. Fostering authentic community participation in research can contribute to the movement for decolonizing public and global health. This can also bring benefits to marginalized communities through interventions that are more relevant to their contexts and needs.

ACKNOWLEDGEMENTS

The authors would like to thank the health librarians Sylvie Fontaine and Viviane Angers at the Université de Montréal for their support in developing this protocol. We would also like to thank members of the CIET/PRAM research lab at McGill University for sharing their expertise with us.

AUTHORS CONTRIBUTIONS

MCGD developed the scoping review protocol, the search strategy and wrote the first version of the manuscript. KZ and NA contributed to the development of the larger project to be conducted in Dhaka, as part of the COESA cluster randomized controlled trial. GF contributed to drafting the scoping review protocol. IS and NA provided expertise on fuzzy cognitive mapping, Harris' discourse analysis and the *Weight of Evidence* approach. All authors read, provided feedback, and approved the final manuscript.

FUNDING

This work was supported by the Fonds de recherche du Québec – Santé (FRQS) through a doctoral research scholarship awarded to the first author. This scoping review is part of a larger project supported by the Canadian Institutes of Health Research through the Project Grant program 201803PJT-400444-RC2-CFCA-120159. These institutions did not play a role in the development of this protocol.

COMPETING INTERESTS

None declared.

LEGENDS OF FIGURES

For peer review only

Figure 1. Example of a fuzzy cognitive map and associated concepts

Figure 2. Steps of the fuzzy cognitive mapping process for the scoping review. The icons represent the tools used in the different steps of the process (i.e., the PRISMA-ScR guidelines, Microsoft Excel, the software CIETmap 2.0). The illustrations below each step represent what each step will look like in practice.

For peer review only

REFERENCES

1 Winchester MS, BeLue R, Oni T, et al. The Pan-University Network for Global Health: framework for collaboration and review of global health needs. *Glob Health* 2016;12(1):13. https://doi.org/10.1186/s12992-016-0151-2.
2 Elsey H, Agyepong I, Huque R, et al. Rethinking health systems in the context of urbanisation: challenges from four rapidly urbanising low-income and middle-income countries. *BMJ Glob Health* 2019;4(3):e001501. https://doi.org/10.1136/bmjgh-2019-001501.
3 Friel S, Akerman M, Hancock T, et al. Addressing the Social and Environmental Determinants of Urban Health Equity: Evidence for Action and a Research Agenda. *J Urban Health* 2011;88(5):860–74. https://doi.org/10.1007/s11524-011-9606-1.
4 Harpham T. Urban health in developing countries: What do we know and where do we go? *Health Place* 2009;15(1):107–16. https://doi.org/doi: 10.1016/j.healthplace.2008.03.004.
5 Birn AE, Pillay Y, Holtz TH. Textbook of Global Health. Fourth edition. New York (NY): Oxford University Press 2017.
6 Affun-Adegbulu C, Adegbulu O. Decolonising Global (Public) Health: from Western universalism to Global pluriversalities. *BMJ Glob Health* 2020;5(8):e002947. https://doi.org/10.1136/bmjgh-2020-002947.
7 Chaudhuri MM, Mkumba L, Raveendran Y, Smith RD. Decolonising global health: beyond 'reformative' roadmaps and towards decolonial thought. *BMJ Glob Health* 2021;6(7):e006371. https://doi.org/10.1136/bmjgh-2021-006371.
8 Gautier L, Karambé Y, Dossou JP, et al. Rethinking development interventions through the lens of decoloniality in sub-Saharan Africa: The case of global health. *Glob Public Health* 2020;0(0):1–14. https://doi.org/10.1080/17441692.2020.1858134.
9 Zarowsky C. Global health research, partnership, and equity: no more business-as-usual. *BMC Int Health Hum Rights* 2011;11(2):S1. https://doi.org/10.1186/1472-698X-11-S2-S1.
10 Khan M, Abimbola S, Aloudat T, et al. Decolonising global health in 2021: a roadmap to move from rhetoric to reform. *BMJ Glob Health* 2021;6(3):e005604. https://doi.org/10.1136/bmjgh-2021-005604.
11 Jagosh J, Macaulay AC, Pluye P, et al. Uncovering the Benefits of Participatory Research: Implications of a Realist Review for Health Research and Practice. *Milbank Q* 2012;90(2):311–46. https://doi.org/10.1111/j.1468-0009.2012.00665.x.
12 Cargo M, Mercer SL. The Value and Challenges of Participatory Research: Strengthening Its Practice. *Annu Rev Public Health* 2008;29(1):325–50. https://doi.org/10.1146/annurev.publhealth.29.091307.083824.
13 Cyril S, Smith BJ, Possamai-Inesedy A, et al. Exploring the role of community engagement in improving the health of disadvantaged populations: a systematic review. *Glob Health Action* 2015;1:29842. https://doi.org/10.3402/gha.v8.29842.
14 O'Mara-Eves A, Brunton G, McDaid D, et al. Community engagement to reduce inequalities in health: a systematic review, meta-analysis and economic analysis. *Public Health Res* 2013;1(4):1–526. https://doi.org/10.3310/phr01040.
15 Sheuya SA. Improving the Health and Lives of People Living in Slums. *Ann N Y Acad Sci* 2008;1136(1):298–306. https://doi.org/10.1196/annals.1425.003.
16 Ahmed S, Meenar M. Just Sustainability in the Global South: A Case Study of the

For peer review only - http://bmjopen.bmj.com/site/about/guidelines.xhtml

Megacity of Dhaka. *J Dev Soc* 2018;34(4):401–24. https://doi.org/10.1177/0169796X18806740.
17 Adams AM, Rashid SF. Mobilising demand for primary health care services among urban slums: insights from a case study in Bangladesh. *Dev Pract* 2021;31(6):726–38. https://doi.org/10.1080/09614524.2020.1862763.
18 Vearey J, Luginaah I, Magitta NF, et al. Urban health in Africa: a critical global public health priority. *BMC Public Health* 2019;19(1):340. https://doi.org/10.1186/s12889-019-6674-8.
19 Grundy J, Wang X, Hirabayashi KC, et al. Health and immunisation services for the urban poor in selected countries of Asia. *Infect Dis Poverty* 2019;8(1):26. https://doi.org/10.1186/s40249-019-0538-4.
20 Otmani del Barrio M, Simard F, Caprara A. Supporting and strengthening research on urban health interventions for the prevention and control of vector-borne and other infectious diseases of poverty: scoping reviews and research gap analysis. *Infect Dis Poverty* 2018;7(1):94. https://doi.org/10.1186/s40249-018-0462-z.
21 Zinszer K, Caprara A, Lima A, et al. Sustainable, healthy cities: protocol of a mixed methods evaluation of a cluster randomized controlled trial for Aedes control in Brazil using a community mobilization approach. *Trials* 2020;21(1):182. https://doi.org/10.1186/s13063-019-3714-8.
22 Dion A, Carini-Gutierrez A, Jimenez V, et al. Weight of Evidence: Participatory Methods and Bayesian Updating to Contextualize Evidence Synthesis in Stakeholders' Knowledge. *J Mix Methods Res* 2021;16(3):281-306. https://doi.org/10.1177/15586898211037412.
23 Sarmiento I, Paredes-Solís S, Dion A, et al. Maternal health and Indigenous traditional midwives in southern Mexico: contextualisation of a scoping review. *BMJ Open* 2021;11(12):e054542. https://doi:10.1136/bmjopen-2021-054542.
24 Peters M, Godfrey C, McInerney P, et al. Chapter 11: Scoping Reviews. In: Aromataris E, Munn Z, eds. JBI Manual for Evidence Synthesis. JBI; 2020 [cited 2022 Feb 5]. https://wiki.jbi.global/display/MANUAL/Chapter+11%3A+Scoping+reviews.
25 Peters MDJ, Godfrey C, McInerney P, et al. Best practice guidance and reporting items for the development of scoping review protocols. *JBI Evid Synth* 2022;20(4):953–68. https://doi.org/10.11124/JBIES-21-00242.
26 Shamseer L, Moher D, Clarke M, et al. Preferred reporting items for systematic review and meta-analysis protocols (PRISMA-P) 2015: elaboration and explanation. *BMJ* 2015;349:g7647. https://doi.org/10.1136/bmj.g7647.
27 Tricco AC, Lillie E, Zarin W, et al. PRISMA Extension for Scoping Reviews (PRISMA-ScR): Checklist and Explanation. *Ann Intern Med* 2018;169(7):467–73. https://doi.org/10.7326/M18-0850.
28 Goodman RA, Bunnell R, Posner SF. What is "community health"? Examining the meaning of an evolving field in public health. *Prev Med* 2014;67:S58–61. https://doi.org/10.1016/j.ypmed.2014.07.028.
29 MacQueen K, McLellan E, Metzger DS, et al. What Is Community? An Evidence-Based Definition for Participatory Public Health. *Am J Public Health* 2001;91(12):1929–38. https://doi.org/10.2105/AJPH.91.12.1929.
30 World Health Organization. The 7th Global Conference on Health Promotion - Community Empowerment. *World Health Organization* 2009 [cited 2022 Aug 5]. https://www.who.int/teams/health-promotion/enhanced-wellbeing/seventh-global-conference/community-empowerment.

31 Brunton G, Thomas J, O'Mara-Eves A, et al. Narratives of community engagement: a systematic review-derived conceptual framework for public health interventions. *BMC Public Health* 2017;17(1):944. https://doi.org/10.1186/s12889-017-4958-4.
32 Draper AK, Hewitt G, Rifkin S. Chasing the dragon: Developing indicators for the assessment of community participation in health programmes. *Soc Sci Med* 2010;71(6):1102–9. https://doi.org/10.1016/j.socscimed.2010.05.016.
33 Key KD, Furr-Holden D, Lewis EY, et al. The Continuum of Community Engagement in Research: A Roadmap for Understanding and Assessing Progress. *Prog Community Health Partnersh Res Educ Action* 2019;13(4):427–34. https://doi.org/10.1353/cpr.2019.0064.
34 Wallerstein N, Duran B. The theoretical, historical, and practice roots of CBPR. In: Wallerstein N, Duran B, Oetzel JG, Minkler M, eds. Community-Based Participatory Research for Health: Advancing Social and Health Equity. Third Edition. San Francisco (US): Jossey-Bass 2008:17–30.
35 The World Bank. World Bank Country and Lending Groups. The World Bank: Working for a World Free from Poverty 2022 [cited 2022 Aug 25]. https://datahelpdesk.worldbank.org/knowledgebase/articles/906519-world-bank-country-and-lending-groups.
36 The Cochrane Collaboration. LMIC Filters. Cochrane Effective Practice and Organisation of Care: Trusted evidence, informed decisions, better health 2022 [cited 2022 Aug 25]. https://epoc.cochrane.org/lmic-filters.
37 Deuskar C. What does "urban" mean?. World Bank Blogs 2015 [cited 2022 Aug 25]. https://blogs.worldbank.org/sustainablecities/what-does-urban-mean.
38 Morris M, Boruff JT, Gore GC. Scoping reviews: establishing the role of the librarian. *J Med Libr Assoc* 2016;104(4):346–53. https://doi.org/10.5195/jmla.2016.156.
39 Veritas Health Innovation. Covidence systematic review software. Melbourne (Australia) 2022. www.covidence.org.
40 Page A. Unnatural and Unequal: Social Determinants of Gender Inequality and Health and Their Impact on Disaster Management Interventions in Bangladesh. University of Ottawa 2015 [cited 2022 Aug 25]. http://ruor.uottawa.ca/handle/10393/33186.
41 Sarmiento I, Cockcroft A, Dion A, et al. Combining conceptual frameworks on maternal health in Indigenous communities - Fuzzy cognitive mapping using participant and operator-independent weighting. *Field Methods* 2022;34(3):223–39. https://doi.org/10.1177/1525822X211070463.
42 Andersson N, Silver H. Fuzzy cognitive mapping: An old tool with new uses in nursing research. *J Adv Nurs* 2019;75(12):3823–30. https://doi.org/10.1111/jan.14192.
43 Gray S, Gray S, De Kok JL, et al. Using fuzzy cognitive mapping as a participatory approach to analyze change, preferred states, and perceived resilience of social-ecological systems. *Ecol Soc* 2015;20(2). https://doi.org/10.5751/ES-07396-200211.
44 Sarmiento I, Paredes-Solis S, Morris M, et al. Factors influencing maternal health in indigenous communities with presence of traditional midwifery in the Americas: protocol for a scoping review. *BMJ Open* 2020;10(10):e037922. https://doi.org/10.1136/bmjopen-2020-037922.
45 Kosko B. Fuzzy cognitive maps. *Int J Man-Mach Stud* 1986;24(1):65–75. https://doi.org/10.1016/S0020-7373(86)80040-2.
46 Kosko B. Hidden patterns in combined and adaptive knowledge networks. *Int J Approx Reason* 1988;2(4):377–93. https://doi.org/10.1016/0888-613X(88)90111-9.

For peer review only

47 Andersson N, Mitchell S. Epidemiological geomatics in evaluation of mine risk education in Afghanistan: introducing population weighted raster maps. *Int J Health Geogr* 2006;5(1):1. https://doi.org/10.1186/1476-072X-5-1.
48 Sarmiento I, Paredes-Solis S, Loutfi D, et al. Fuzzy cognitive mapping and soft models of indigenous knowledge on maternal health in Guerrero, Mexico. *BMC Med Res Methodol* 2020;20(1):125. https://doi.org/10.1186/s12874-020-00998-w.
49 Niesink P, Poulin K, Šajna M. Computing transitive closure of bipolar weighted digraphs. *Discrete Appl Math* 2013;161(1):217–43. https://doi.org/10.1016/j.dam.2012.06.013.
50 Harris ZS. Discourse Analysis. *Language* 1952;28(1):1–30. https://doi.org/10.2307/409987.
51 Arksey H, O'Malley L. Scoping studies: towards a methodological framework. *Int J Soc Res Methodol* 2005;8(1):19–32. https://doi.org/10.1080/1364557032000119616.
52 Boyko JA, Lavis JN, Abelson J, et al. Deliberative dialogues as a mechanism for knowledge translation and exchange in health systems decision-making. *Soc Sci Med* 2012;75(11):1938–45. https://doi.org/10.1016/j.socscimed.2012.06.016.
53 Dion A, Nakajima A, McGee A, et al. How Adolescent Mothers Interpret and Prioritize Evidence About Perinatal Child Protection Involvement: Participatory Contextualization of Published Evidence. *Child Adolesc Soc Work J* 2022. https://doi.org/10.1007/s10560-022-00865-1.

For peer review only

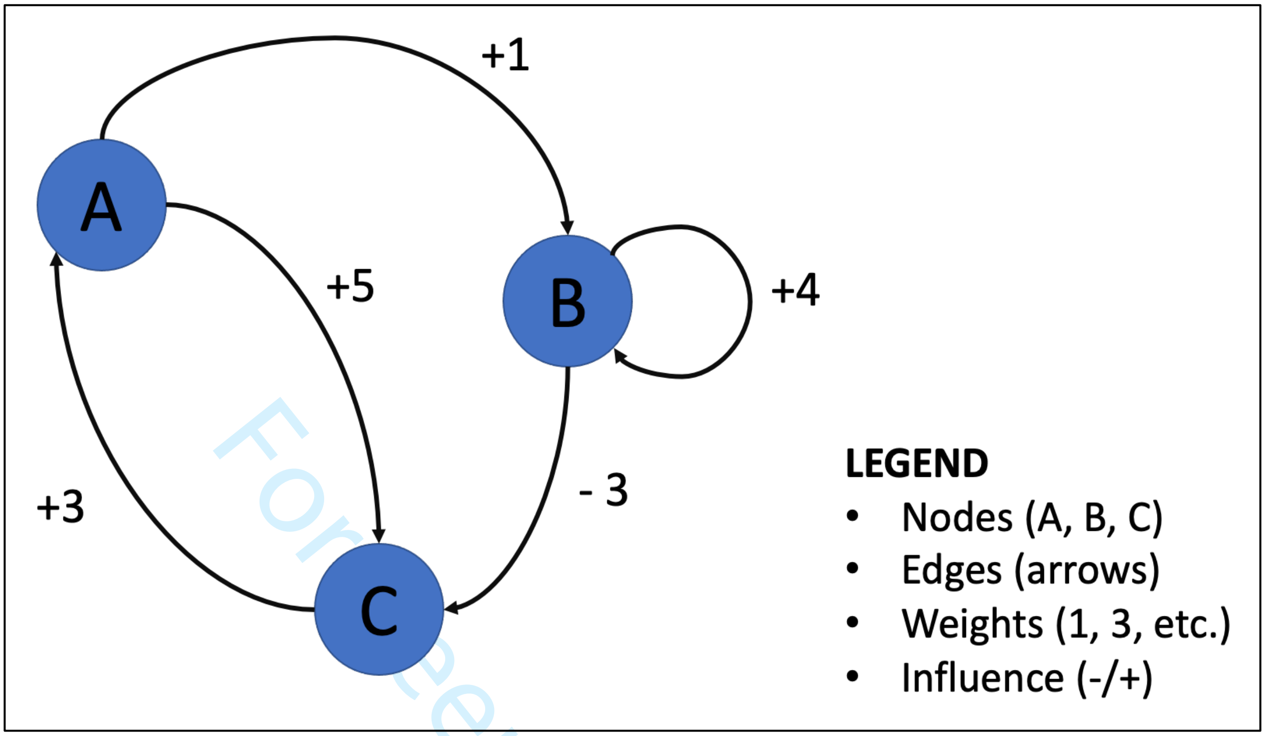

For peer review only

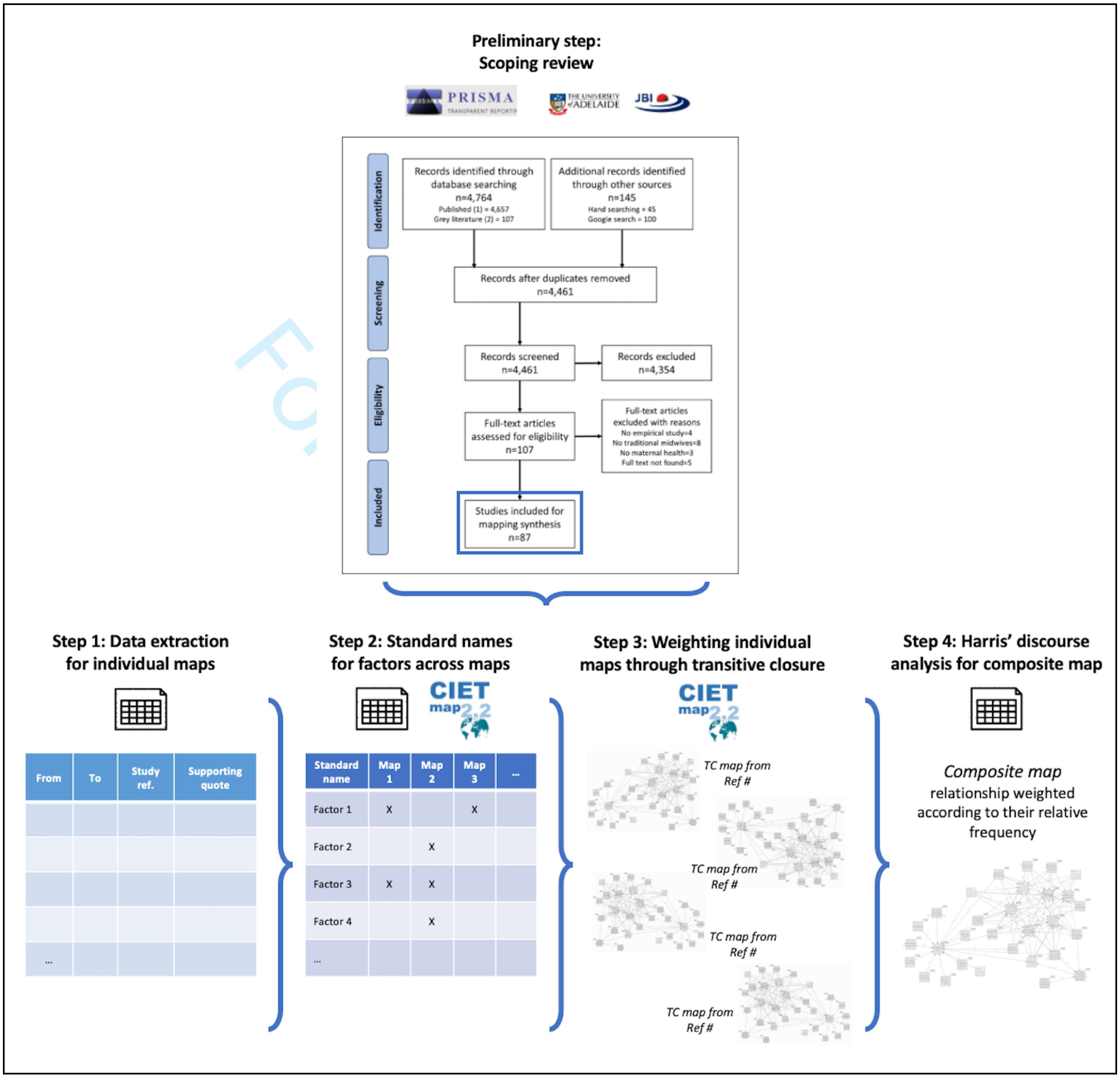

Preliminary step:
Scoping review
PRISMA
THE UNIVERSITY of ADELAIDE
JBI
Identification
Screening
Eligibility
Included
Records identified through database searching n=4,764
Published (1) = 4,657
Grey literature (2) = 107
Additional records identified through other sources n=145
Hand searching = 45
Google search = 100
Records after duplicates removed n=4,461
Records screened n=4,461
Records excluded n=4,354
Full-text articles assessed for eligibility n=107
Full-text articles excluded with reasons
No empirical study=4
No traditional midwives=8
No maternal health=3
Full text not found=5
Studies included for mapping synthesis n=87
Step 1: Data extraction for individual maps
From
To
Study ref.
Supporting quote
...
Step 2: Standard names for factors across maps
CIET map2.2
Standard name
Map 1
Map 2
Map 3
...
Factor 1
Factor 2
Factor 3
Factor 4
X
Step 3: Weighting individual maps through transitive closure
CIET map2.2
TC map from Ref #
Step 4: Harris' discourse analysis for composite map
Composite map
relationship weighted according to their relative frequency

PRISMA-P 2015 Checklist

This checklist has been adapted for use with protocol submissions to *Systematic Reviews* from Table 3 in Moher D et al: Preferred reporting items for systematic review and meta-analysis protocols (PRISMA-P) 2015 statement. *Systematic Reviews* 2015 **4**:1

Section/topic	#	Checklist item	Information reported		Line number(s)
			Yes	No	
**ADMINISTRATIVE INFORMATION**					
**Title**					
Identification	1a	Identify the report as a protocol of a systematic review	☒	☐	Page 1
Update	1b	If the protocol is for an update of a previous systematic review, identify as such	☐	☒	N/A
**Registration**	2	If registered, provide the name of the registry (e.g., PROSPERO) and registration number in the Abstract	☐	☒	N/A
**Authors**					
Contact	3a	Provide name, institutional affiliation, and e-mail address of all protocol authors; provide physical mailing address of corresponding author	☒	☐	Page 1
Contributions	3b	Describe contributions of protocol authors and identify the guarantor of the review	☒	☐	Page 12
**Amendments**	4	If the protocol represents an amendment of a previously completed or published protocol, identify as such and list changes; otherwise, state plan for documenting important protocol amendments	☐	☒	N/A
**Support**					
Sources	5a	Indicate sources of financial or other support for the review	☒	☐	Page 12
Sponsor	5b	Provide name for the review funder and/or sponsor	☒	☐	Page 12
Role of sponsor/funder	5c	Describe roles of funder(s), sponsor(s), and/or institution(s), if any, in developing the protocol	☒	☐	Page 12
**INTRODUCTION**					
**Rationale**	6	Describe the rationale for the review in the context of what is already known	☒	☐	Page 3 (bottom)
**Objectives**	7	Provide an explicit statement of the question(s) the review will address with reference to participants, interventions, comparators, and outcomes (PICO)	☒	☐	Page 4 (bottom)
**METHODS**					

For peer review only

BioMed Central
The Open Access Publisher

Section/topic	#	Checklist item	Information reported Yes	No	Line number(s)
**Eligibility criteria**	8	Specify the study characteristics (e.g., PICO, study design, setting, time frame) and report characteristics (e.g., years considered, language, publication status) to be used as criteria for eligibility for the review	☒	☐	Pages 5-6
**Information sources**	9	Describe all intended information sources (e.g., electronic databases, contact with study authors, trial registers, or other grey literature sources) with planned dates of coverage	☒	☐	Page 5 (top); Page 6 (bottom)
**Search strategy**	10	Present draft of search strategy to be used for at least one electronic database, including planned limits, such that it could be repeated	☒	☐	Pages 6-8
STUDY RECORDS					
Data management	11a	Describe the mechanism(s) that will be used to manage records and data throughout the review	☒	☐	Page 8
Selection process	11b	State the process that will be used for selecting studies (e.g., two independent reviewers) through each phase of the review (i.e., screening, eligibility, and inclusion in meta-analysis)	☒	☐	Page 8
Data collection process	11c	Describe planned method of extracting data from reports (e.g., piloting forms, done independently, in duplicate), any processes for obtaining and confirming data from investigators	☒	☐	Pages 8-9
**Data items**	12	List and define all variables for which data will be sought (e.g., PICO items, funding sources), any pre-planned data assumptions and simplifications	☒	☐	Page 9 (point e)
**Outcomes and prioritization**	13	List and define all outcomes for which data will be sought, including prioritization of main and additional outcomes, with rationale	☒	☐	Pages 8-9 (points of data extraction form)
**Risk of bias in individual studies**	14	Describe anticipated methods for assessing risk of bias of individual studies, including whether this will be done at the outcome or study level, or both; state how this information will be used in data synthesis	☐	☒	N/A
DATA					
**Synthesis**	15a	Describe criteria under which study data will be quantitatively synthesized	☐	☒	N/A
	15b	If data are appropriate for quantitative synthesis, describe planned summary measures, methods of handling data, and methods of combining data from studies, including any planned exploration of consistency (e.g., I^2, Kendall's tau)	☐	☒	N/A
	15c	Describe any proposed additional analyses (e.g., sensitivity or subgroup analyses, meta-regression)	☐	☒	N/A
	15d	If quantitative synthesis is not appropriate, describe the type of summary planned	☒	☐	Pages 9-10
**Meta-bias(es)**	16	Specify any planned assessment of meta-bias(es) (e.g., publication bias across studies, selective reporting within studies)	☐	☒	N/A
**Confidence in cumulative evidence**	17	Describe how the strength of the body of evidence will be assessed (e.g., GRADE)	☐	☒	N/A

For peer review only

For peer review only

BioMed Central
The Open Access Publisher

BMJ Open

Why urban communities from low- and middle-income countries participate in public and global health research: Protocol for a scoping review

Journal:	*BMJ Open*
Manuscript ID	bmjopen-2022-069340.R1
Article Type:	Protocol
Date Submitted by the Author:	21-Mar-2023
Complete List of Authors:	Gagnon-Dufresne, Marie-Catherine; University of Montreal School of Public Health, Department of Social and Preventive Medicine; Centre de recherche en santé publique (CReSP)<br>Sarmiento, Ivan; McGill University, Department of Family Medicine; Universidad Del Rosario, Escuela de Medicina y Ciencias de la Salud<br>Fortin, Geneviève; University of Montreal, School of Public Health; Centre de recherche en santé publique (CReSP)<br>Andersson, Neil; McGill University, Department of Family Medicine; Universidad Autonoma de Guerrero - Campus Acapulco, Centro de Investigación de Enfermedades Tropicales (CIET)<br>Zinszer, Kate; University of Montreal School of Public Health, Department of Social and Preventive Medicine; Centre de recherche en santé publique (CReSP)
**Primary Subject Heading**:	Public health
Secondary Subject Heading:	Global health, Public health
Keywords:	PUBLIC HEALTH, PREVENTIVE MEDICINE, QUALITATIVE RESEARCH

SCHOLARONE™
Manuscripts

Why urban communities from low- and middle-income countries participate in public and global health research: Protocol for a scoping review

CORRESPONDING AUTHOR

Marie-Catherine Gagnon-Dufresne
École de santé publique, Université de Montréal
7101 Park Avenue, Montréal, QC
Canada H3N 1X9
marie-catherine.gagnon-dufresne@umontreal.ca

AUTHORS

Marie-Catherine Gagnon-Dufresne[1,2,3*], Iván Sarmiento[3,4], Geneviève Fortin[1,2], Neil Andersson[3,5], Kate Zinszer[1,2]

1. École de santé publique, Université de Montréal, Montréal (Canada)
2. Centre de recherche en santé publique (CReSP), Montréal (Canada)
3. CIET-PRAM, Department of Family Medicine, McGill University, Montréal (Canada)
4. Grupo de Estudios en Sistemas Tradicionales de Salud, Escuela de Medicina y Ciencias de la Salud, Universidad del Rosario, Bogotá (Colombia)
5. Centro de Investigación de Enfermedades Tropicales (CIET), Universidad Autónoma de Guerrero, Acapulco (Mexico)

WORD COUNT

3,786 words

For peer review only

Why urban communities from low- and middle-income countries participate in public and global health research: Protocol for a scoping review

ABSTRACT

Introduction: As the number of people living in cities increases worldwide, particularly in low- and middle-income countries (LMICs), urban health is a growing priority of public and global health. Rapid unplanned urbanization in LMICs has exacerbated inequalities, putting the urban poor at increasing risk of ill health due to difficult living conditions in cities. Collaboration with communities in research is a key strategy for addressing the challenges they face. The objective of this scoping review is therefore to identify factors that influence the participation of urban communities from LMICs in public and global health research.

Methods and analysis: We will develop a search strategy with a health librarian to explore the following databases: MEDLINE, Embase, Web of Science, Cochrane, Global Health, and CINAHL. We will use MeSH terms and keywords exploring the concepts of "low- and middle-income countries", "community participation in research", and "urban settings" to look at empirical research conducted in French or English. There will be no restriction in terms of dates of publication. Two independent reviewers will screen and select studies, first based on titles and abstracts, and then on full text. Two reviewers will extract data. We will summarize the results using tables and fuzzy cognitive mapping.

Ethics and dissemination: This scoping review is part of a larger project to be approved by the University of Montréal's Research Ethics Committee for Science and Health in Montréal (Canada), and the Institutional Review Board of the James P. Grant School of Public Health at BRAC University in Dhaka (Bangladesh). Results from the review will contribute to a participatory process seeking to combine scientific evidence with experiential knowledge of stakeholders in Dhaka to understand how to better collaborate with communities for research. The review could contribute to a shift towards research that is more inclusive and beneficial for communities.

KEYWORDS

public health, global health, community-based participatory research, stakeholder participation, urban population, urban health, fuzzy logic

STRENGTHS AND LIMITATIONS OF THIS STUDY

- What constitutes 'communities', 'participation in research' and 'urban settings' can be defined in various ways, so it will be crucial to highlight how these concepts are defined in the literature included in the scoping review.

For peer review only

- Reporting on community participation is heterogeneous, and identifying the research approaches, health issues, contexts, and community characteristics that favor participation will be challenging.
- The scoping review will summarize results using fuzzy cognitive mapping, providing soft models of causality that can be contextualized in the experience of local stakeholders in Dhaka.
- The methods presented in this scoping review protocol could be replicated to compare and combine scientific evidence and experiential knowledge anywhere.

INTRODUCTION

As the number of people living in cities increases worldwide, particularly in low- and middle-income countries (LMICs), the health of urban populations is a growing priority of public and global health.[1] Urbanization brings changes to the disease burdens, determinants of health, and patterns of health inequalities.[1,2] Despite the benefits of urban living and progress in population health, rapid and unplanned urbanization in LMICs has worsened health inequalities.[3] Not everyone in cities experiences these improvements equally, as policies and other efforts often fail to reach the most marginalized communities, including those living in informal settlements.[3] The urban poor are therefore at increased risk of ill health due to the difficult living conditions in cities.[3,4]

Several researchers in public and global health have criticized the reproduction of colonial relations in efforts to improve the health of populations in LMICs, as these efforts are often led by foreign researchers with little input from local populations.[5–7] The resulting unequal power dynamics between researchers and communities are among the reasons research makes little or mixed contributions to health.[8] In response, there are increasing calls to decolonize public and global health through community participation in research, to better meet their needs and ensure local relevance of the initiatives put in place to improve their health.[8–10]

Community participation in health research is recognized for building capacity and fostering conditions to enable better community control over determinants of their health.[11,12] Community participation can lead to equitable partnerships between communities and researchers, making research more empowering and effective.[12] However, most health research uses top-down community engagement approaches rather than bottom-up participatory methods.[13] While there is no standard definition of community participation, different uses of the term form a continuum from consulting or informing communities to sharing power with them.[13,14]

Some of the world's most populated cities are located in South Asian countries, including Bangladesh, India, and Pakistan.[15] These countries are characterized by high levels of urban poverty, with more than 50% of their urban population estimated to be living in informal settlements.[16,17] Considering that urbanization in these contexts is inextricably linked to complex patterns of discrimination and social exclusion for residents of informal

For peer review only

settlements, it is crucial that public/global health professionals and researchers collaborate with these communities to understand their health priorities and find innovative solutions to improve their health [18,19].
Collaborating with marginalized urban communities is a key strategy for addressing the many challenges they face.[3,20,21] Yet, these communities represent a particularly hard-to-reach group, as asymmetries in access to resources and opportunities affect their capacity to fully participate in and benefit from research implement to improve their health.[20,22] There is therefore an urgent need to better understand the barriers and enablers to their participation.[23–25]

The objective of this scoping review is to identify factors that influence the participation of urban communities from low- and middle-income countries in public and global health research. Part of a larger project, this scoping review will contribute to a dialogue between scientific and experiential knowledge on the factors that influence community participation in public and global health research. We will contextualize the results from the scoping review in the views of stakeholders in Dhaka (Bangladesh) in a participatory process to reflect their experiences. This contextualization will identify barriers and enablers to participation that are specific to Dhaka, in preparation for a cluster randomized controlled trial testing the effect of a participatory community mobilization intervention for reducing dengue infection.[26]

METHODS AND ANALYSIS

This review is part of a larger project aimed at comparing and combining different knowledge sources to provide a knowledge base for decision making, that will be used to inform a cluster randomized controlled trial to reduce dengue infection in Dhaka.[26] The larger project will consider four knowledge sources: 1) the scoping review described in this protocol; 2) the views of Canadian and Bangladeshi public and global health researchers; 3) the views of personnel from community-based organizations in Dhaka; 4) the views of community members from underserved neighbourhoods in Dhaka. We will adapt the *Weight of Evidence* approach [27,28] and use fuzzy cognitive mapping to bring these different knowledge sources into conversation.

In this protocol, we focus on describing the procedures to conduct the scoping review and briefly discuss how the results will be used to inform the subsequent phases of the larger project. The proposed scoping review will be conducted in accordance with the PRISMA-ScR (Preferred Reporting Items for Systematic Reviews and Meta-Analyses – Extension for Scoping Reviews) guidelines and Joanna Briggs Institute's methodology to ensure accuracy, completeness and transparency.[29–32]

Review questions

This scoping review will seek to answer the following question, developed according to the PCC method (Participants, Concept, Context) recommended by the Joanna Briggs Institute [29]: What factors influence the participation of urban communities from low- and middle-income countries in research, based on evidence from the public and global health literature?

For peer review only - http://bmjopen.bmj.com/site/about/guidelines.xhtml

The review will also seek to answer the following sub-questions:

1) What are the main barriers and enablers of community participation in public and global health research?
2) What is the relative influence of these factors on community participation?
3) What research approaches are most and least favourable to community participation?
4) What public and global health issues are most and least favourable to community participation?
5) What contexts are most and least favourable to community participation?
6) What community characteristics are most and least favourable to community participation?

Eligibility criteria

Types of sources

This scoping review will consider empirical studies with quantitative, qualitative, and mixed methods designs. We will not include literature reviews and meta-analyses, but we will consider including the empirical studies reported in reviews and meta-analyses if relevant. We will not consider grey or scientific literature reporting on programs, policy or other initiative implemented outside of research purposes.

Participants

This review will look at research participation at the community level rather than at the individual level, therefore excluding studies discussing the participation of individuals in research (*i.e.*, patient engagement, individual motivation to participate). Communities refer to population groups and the locus (*i.e.*, place, venue, or other units) of their actions.[33] Communities can be understood as groups of people with diverse characteristics that are linked by social ties or identities; share common interests or concerns; and engage in joint action in settings, venues or areas that may be physically, geographically, culturally or politically defined.[33–35] The definition of what constitutes a community remains broad for this scoping review but will focus on urban communities in low- and middle-income countries. This is justified by the fact that the larger project is part of a cluster randomized controlled trial on dengue which will be conducted in Dhaka (Bangladesh), and that the factors influencing community participation in research will likely vary between rural and urban communities, and between high- and low- and middle-income countries.

Concept

Community-engaged research is a broad topic, defined in various ways and used for various reasons. It is often an umbrella term for research involving the participation of non-academic stakeholders, with diverse models and conceptual frameworks.[14] There is no standard definition of community participation in research in public and global health.[13,14] The distinction between 'engagement,' 'mobilization' and 'participation' in research is unclear, as these terms are often used interchangeably and with changing definitions.

For peer review only

Various authors discuss the levels of community participation in research as being positioned along a continuum, ranging from information provision and exchange, to consultation, to co-production, and to shared leadership and community control.[14,36–39] For this scoping review, we will not restrict the search to a specific level of participation, but we will examine and compare how different approaches (i.e., community mobilization interventions, partnered research, community-based participatory research designs, etc.) are found to enable or hinder participation. However, research in which there is little community involvement (*i.e.*, health education, consultation efforts in which communities have no decision-making power over some aspects of the research) will be excluded.

Context

This review will focus on communities located in urban settings in low- and middle-income countries. The definition of low- and middle-income countries used for the review will use the World Bank's classification from the 2023 fiscal year, based on countries' gross national incomes per capita.[40] The Cochrane Effective Practice and Organisation of Care (EPOC) group has developed a filter for literature reviews based on the World Bank classification to identify studies relevant to LMICs.[41]

There is no standard international definition of what constitutes an urban setting. Each country has its own definition, following nationally defined criteria on population size, population density, type of economic activity, physical characteristics, level of infrastructure, or other characteristics.[42] Considering the lack of a common definition, the scoping review will consider all studies in LMICs conducted in urban settings or cities as identified by the authors, including neighbourhoods and informal settlements (slums) in cities.

Exclusion criteria

To ensure the selection of relevant studies for the review, we will use the following exclusion criteria:

- Grey literature
- Not empirical research
- Discussing community engagement, participation, partnership, or mobilization in contexts other than research (*i.e.*, programs, policy, urban planning)
- Focused on individual engagement in research (*i.e.*, patient engagement, individual motivation)
- Not discussing factors that influence community participation in research
- Conducted in contexts other than urban settings
- Conducted in countries other than low- and middle-income countries
- Full text of the reference is not available

Table 1 summarizes the inclusion and exclusion criteria used to select articles for the scoping review.

Table 1. Inclusion and exclusion criteria for the scoping review

Inclusion criteria	Exclusion criteria

For peer review only

• Empirical qualitative, quantitative, and mixed methods research<br>• Discussing community engagement, participation, partnership or mobilization in research<br>• Focused on community-level engagement<br>• Discussing factors that influence community participation in research<br>• Conducted in urban settings<br>• Conducted in low- and middle-income countries	• Not empirical research<br>• Grey literature<br>• Discussing community engagement, participation, partnership, or mobilization in contexts other than research<br>• Focused on individual-level engagement<br>• Not discussing factors that influence community participation in research<br>• Conducted in contexts other than urban settings<br>• Conducted in countries other than low- and middle-income countries<br>• Full text of the reference is not available

Search strategy

The search strategy will be developed with the help of a health librarian. It will explore the following databases: MEDLINE, Embase, Web of Science, Cochrane, Global Health, and CINAHL. We will use MeSH terms and keywords to identify studies reported in English or French. We will not have restrictions in terms of dates of publication. We will not contact the authors of the articles selected to request additional information. Table 2 presents the initial search strategy for MEDLINE, which will be adapted for each database.

Table 2. Example of a potential search strategy for MEDLINE

**Concept 1: Low- and middle-income countries**
1. (afghanistan or albania or algeria or american samoa or angola or "antigua and barbuda" or antigua or barbuda or argentina or armenia or armenian or aruba or azerbaijan or bahrain or bangladesh or barbados or "republic of Belarus" or belarus or byelarus or belorussia or byelorussian or belize or british honduras or benin or dahomey or bhutan or bolivia or "bosnia and herzegovina" or bosnia or herzegovina or botswana or bechuanaland or brazil or brasil or bulgaria or burkina faso or burkina fasso or upper volta or burundi or urundi or cabo verde or cape verde or cambodia or kampuchea or khmer republic or cameroon or cameron or cameroun or central african republic or ubangi shari or chad or chile or china or colombia or comoros or comoro islands or iles comores or mayotte or "democratic republic of the congo" or democratic republic congo or congo or zaire or costa rica or "cote d'ivoire" or "cote d' ivoire" or cote divoire or cote d ivoire or ivory coast or croatia or cuba or cyprus or czech republic or czechoslovakia or djibouti or french somaliland or dominica or dominican republic or ecuador or egypt or united arab republic or el salvador or equatorial guinea or spanish guinea or eritrea or estonia or eswatini or swaziland or ethiopia or fiji or gabon or gabonese republic or gambia or "georgia (republic)" or georgian or ghana or gold coast or gibraltar or greece or grenada or guam or guatemala or guinea or guinea bissau or guyana or british guiana or haiti or hispaniola or honduras or hungary or india or indonesia or timor or iran or iraq or "isle of man" or jamaica or jordan or kazakhstan or kazakh or kenya or "democratic people's republic of korea" or "republic of korea" or north korea or south korea or korea or kosovo or kyrgyzstan or kirghizia or kirgizstan or kyrgyz republic or kirghiz or laos or lao pdr or "lao people's democratic republic" or latvia or lebanon or lebanese republic or lesotho or basutoland or liberia or libya or libyan arab jamahiriya or lithuania or macau or macao or "republic of north macedonia" or macedonia or madagascar or malagasy republic or malawi or nyasaland or malaysia or malay federation or malaya federation or maldives or indian ocean islands or indian ocean or mali or malta or micronesia or "federated states of Micronesia" or kiribati or marshall islands or nauru or northern mariana islands or palau or tuvalu or mauritania or mauritius or mexico or moldova or moldovian or mongolia or montenegro or morocco or ifni or mozambique or portuguese east africa or myanmar or burma or namibia or nepal or netherlands antilles or nicaragua or niger or nigeria or oman or muscat or pakistan or panama or papua new guinea or new guinea or paraguay or peru or philippines or philipines or phillipines or phillippines or poland or "polish people's republic" or portugal or portuguese republic or puerto rico or romania or russia or russian federation or ussr or soviet union or "union of soviet socialist republics" or rwanda or ruanda or samoa or

For peer review only

pacific islands or polynesia or samoan islands or navigator island or navigator islands or "sao tome and principe" or saudi arabia or senegal or serbia or seychelles or sierra leone or slovakia or slovak republic or slovenia or melanesia or solomon island or solomon islands or norfolk island or norfolk islands or somalia or south africa or south sudan or sri lanka or ceylon or "saint kitts and nevis" or "st. kitts and nevis" or saint lucia or "st. lucia" or "saint vincent and the grenadines" or saint vincent or "st. vincent" or grenadines or sudan or suriname or surinam or dutch guiana or netherlands guiana or syria or syrian arab republic or tajikistan or tadjikistan or tadzhikistan or tadzhik or tanzania or tanganyika or thailand or siam or timor leste or east timor or togo or togolese republic or tonga or "trinidad and tobago" or trinidad or tobago or tunisia or turkey or turkmenistan or turkmen or uganda or ukraine or uruguay or uzbekistan or uzbek or vanuatu or new hebrides or venezuela or vietnam or viet nam or middle east or west bank or gaza or palestine or yemen or yugoslavia or zambia or zimbabwe or northern rhodesia or global south or "africa south of the sahara" or sub-saharan africa or subsaharan africa or africa, central or central africa or africa, northern or north africa or northern africa or magreb or maghrib or sahara or africa, southern or southern africa or africa, eastern or east africa or eastern africa or africa, western or west africa or western africa or west indies or indian ocean islands or caribbean or central america or latin america or "south and central america" or south america or asia, central or central asia or asia, northern or north asia or northern asia or asia, southeastern or southeastern asia or south eastern asia or southeast asia or south east asia or asia, western or western asia or europe, eastern or east europe or eastern europe or developing country or developing countries or developing nation? or developing population? or developing world or less developed countr* or less developed nation? or less developed population? or less developed world or lesser developed countr* or lesser developed nation? or lesser developed population? or lesser developed world or under developed countr* or under developed nation? or under developed population? or under developed world or underdeveloped countr* or underdeveloped nation? or underdeveloped population? or underdeveloped world or middle income countr* or middle income nation? or middle income population? or low income countr* or low income nation? or low income population? or lower income countr* or lower income nation? or lower income population? or underserved countr* or underserved nation? or underserved population? or underserved world or under served countr* or under served nation? or under served population? or under served world or deprived countr* or deprived nation? or deprived population? or deprived world or poor countr* or poor nation? or poor population? or poor world or poorer countr* or poorer nation? or poorer population? or poorer world or developing econom* or less developed econom* or lesser developed econom* or under developed econom* or underdeveloped econom* or middle income econom* or low income econom* or lower income econom* or low gdp or low gnp or low gross domestic or low gross national or lower gdp or lower gnp or lower gross domestic or lower gross national or lmic or lmics or third world or lami countr* or transitional countr* or emerging economies or emerging nation?).ti,ab,sh,kf.<br>2. Developing countries/<br>3. 1 or 2
**Concept 2: Community participation in research**
4. (((participat* or communit* or partner*) adj3 research) or (communit* adj3 (participat* or engage* or mobili?ation or intervention*)) or participatory or CBPR).ti,ab,sh,kf.<br>5. Community-based participatory research/<br>6. Community participation/<br>7. 4 or 5 or 6
**Concept 3: Urban settings**
8. (urban* or city or cities or metropol* or megacit* or megalop* or municipalit* or "informal settlement" or "informal settlements" or slum* or favela* or "shanty town" or "shanty towns" or ghetto* or bustee*).ti,ab,sh,kf.<br>9. Urban Health/<br>10. Urban Population/<br>11. Cities/<br>12. Urbanization/<br>13. Poverty Areas/<br>14. 8 or 9 or 10 or 11 or 12 or 13
**Final search strategy**
15. 3 and 7 and 14

For peer review only

The search strategy will be developed with the input from a librarian and the research team to identify new keywords.[43] After our initial screening in MEDLINE, we will search the included articles for new keywords. A new search will then be conducted combining the newly found MeSH terms and keywords to the existing search. A librarian will assess whether these new terms should be included in the final search strategy. When all articles are screened, we will search the reference lists of selected studies to identify additional studies meeting our inclusion criteria.

Study selection

Following the search, all identified citations will be collated and uploaded into Covidence,[44] and duplicates will be removed. Study selection will be conducted in two phases by two independent reviewers, who will reconcile differences by consensus. A third independent reviewer will help resolve any further disagreement.

The initial screening of the retrieved sources will use titles and abstracts. The second phase of selection will use full text. Reasons for excluding sources at full text that do not meet the inclusion criteria will be recorded and reported in the review. The results of the search and the study selection process will be reported in the flow diagram developed by PRISMA-ScR.[32,45]

Because the aim of scoping reviews is to map the available evidence on a specific topic, we will not perform an assessment of the methodological quality or risk of bias of the articles included in the review [29]. However, the data extraction form will report the research design as well as the data collection and analysis methods of selected articles. This will allow us to dress a portrait of the available evidence on the factors influencing the participation of urban communities in research.

Data extraction

Two reviewers will develop and pilot a data extraction form, and extract the data in Covidence.[44] The form will include:

a. Details on the study (title, names of the authors, year of publication, study objectives, research design, and data collection and analysis methods)
b. The country and the urban contexts in which studies were conducted
c. The communities targeted
d. If available, the definitions of ‘community’ used by the authors
e. The participation approach used and the extent of community participation
f. The findings regarding the factors (barriers, enablers, and other factors) influencing the participation of urban communities in public and global health research
g. If available, the relative influence (qualitative or quantitative) of the factors identified on community participation
h. If available, other relations among the factors identified, and their relative influence on community participation
i. Explanation of the relationships between factors (quotes from the articles)

For peer review only

We will not systematically extract data on the results of the studies since this is outside the scope of the review objectives and research question.

The data extraction form will be piloted before beginning the study selection process with a random sample of 5 studies among all the studies to be reviewed. The pilot test will help identify missing data and will contribute to ensuring that the reporting of participation approaches and factors influencing community participation is coherent across studies and between the two reviewers. The data extraction form will be modified and revised as necessary, in an iterative manner, during the data extraction process. Modifications will be detailed in the report of the review.

Any disagreements on data extraction that arise between the two reviewers at the pilot or data extraction stages will be resolved by consensus, or by discussion with a third independent reviewer if necessary.

Data analysis and presentation

The presentation of results will follow the PRISMA-ScR guidelines.[32] We will present the results in tables and use fuzzy cognitive mapping (FCM) to illustrate how the different factors identified influence community participation in research, adapting the *Weight of Evidence* approach.[27,46] A narrative summary will also accompany the tabulated and mapped results, describing how the results relate to the review objectives and questions.

FCM uses graph theory and fuzzy logic to generate soft models of how change could happen based on assumed causal relationships.[47–49] These soft models are illustrated through graphs called fuzzy cognitive maps (Figure 1), which are used to represent assumed causal relationships between concepts.[50,47] The maps use nodes (factors affecting the issue) and edges (arrows representing the relationships between factors), weighted by the relative strength of their influence on the issue of interest.[51,47,27] Depending on the knowledge source of the maps, edges can have different values (hence the term *fuzzy*) to quantify their influence in a relative way.[49]

FCM will be the cornerstone for the presentation of the scoping review, through the creation of fuzzy cognitive maps to represent: 1) each article included in the review; and 2) a composite map for the whole review. FCM will allow to summarize in a composite map the relative influence that each factor might have on community participation, in relation to all the other factors identified in the review.[27,28,47] We will go through several steps (detailed below) to create the composite literature-based fuzzy cognitive map of the barriers and enablers to community participation (Figure 2).

First, we will create one fuzzy cognitive map for each article selected in the scoping review (Step 1 in Figure 2). In each individual map, community participation will be the outcome of interest. We will include each barrier and enabler of community participation mentioned in the article (point e in the data extraction form) as a node in the map, which we will organize in a table. This table will have two initial columns indicating the origin factor (from) and the consequence factor (to). Additional columns will present the evidence supporting

For peer review only

the relationship between both factors from the article (point h in the data extraction form). Each relationship identified will be a row in the table.[49]

Second, once all the individual tables are created, we will standardize the names of the factors across the individual articles so that they can be comparable (Step 2 in Figure 2).[46] On each individual map reporting the relationships identified in each study, we will calculate fuzzy transitive closure in the open access CIETmap 2.0 (Step 3 in Figure 2).[52] Fuzzy transitive closure is a mathematical model used to calculate the influence of each relationship on community participation, considering all the possible relationships represented in the map.[53,54] After transitive closure, each relationship will have a value between 0 (having no influence) and 1 (having the strongest influence) to represent the relative strength of their influence on community participation, with positive and negative signs indicating whether the relationship is stimulative or inhibitive.[27,53]

Third, we will create a composite map for the whole review (Step 4 in Figure 2). To create this composite map, we will attribute weights to each node using Harris' discourse analysis, an analytical approach developed in the 1950s based the frequency of occurrence of discourse elements sharing similar meanings in a body of text, such as a literature review.[55,46] We will consider the frequency of occurrence of each relationship across the maps developed for each article in the scoping review. This means that a factor that is repeated in multiple maps would have a stronger causal meaning for community participation than a factor only mentioned in one or two maps.[46] We will establish the relative frequency of factors by dividing each occurrence by the highest frequency across all the maps. We will therefore obtain a value between 0 for the relationships that did not exist and 1 for the relationship most frequently mentioned.[46] These different steps will allow us to create a composite map representing all the factors identified in the scoping review, weighted according to their relative frequency.

Patient and public involvement

We will include a consultation phase in the scoping review, as Arksey & O'Malley (2005) recognize the benefit of discussing the results of a review with experts.[56] The *Weight of Evidence* approach, which we will adapt for this scoping review, advocates for experiential knowledge to be considered on an equal footing with the evidence synthesized from the literature.[27] Therefore, in the context of the larger project, which adopts a participatory methodology and involves a community advisory board, people concerned with the issue of interest (*i.e.*, participation of urban communities in health research) will be invited to contextualize the scoping review.

After conducting the scoping review, stakeholders in Dhaka will develop their own fuzzy cognitive maps on the factors that they believe can influence community participation in the Bangladeshi context. After developing their maps, they will interpret the literature-based map from the scoping review by comparing the results with their own maps. We will seek the perspectives of three stakeholder groups, namely public and global health researchers, community-based organizations, and community stakeholders.

For peer review only

Finally, we will use the composite map from the scoping review and the various maps from these three stakeholder groups to generate a final map incorporating these two knowledge sources. The literature-based map, the stakeholder maps and this final map will be reviewed through deliberative dialogue with stakeholders in Dhaka.[57] The maps and discussions with stakeholders will inform decision-making for the cluster randomized controlled trial on dengue testing a participatory community mobilization intervention, where communities in Dhaka will develop their own solutions to reduce dengue infection. These steps will be conducted and reported separately.

ETHICS AND DISSEMINATION

This scoping review does not require ethics approval. However, the consultation process is part of a larger project which will need to be approved by the University of Montréal's Research Ethics Committee for Science and Health in Montréal (Canada), and the Institutional Review Board of the James P. Grant School of Public Health at BRAC University in Dhaka (Bangladesh). We will apply for ethics approval for the larger project at both universities by August 2023. We will share the results from the scoping review with the scientific community through scientific articles and presentations at conferences, and with local stakeholders in Dhaka through a participatory process involving fuzzy cognitive mapping and deliberative dialogue. Results from this process will directly inform the implementation of the cluster randomized controlled trial on dengue in Dhaka.[26]

DISCUSSION

This protocol described a scoping review which will seek to identify and map the factors that can influence the participation of urban communities from low- and middle-income countries in public and global health research. The review will contribute to the understanding of how to foster the participation of these communities in research, so that it can better respond to local needs. Given that marginalized urban communities represent a particularly hard-to-reach group in research and that urban health is a growing priority of public and global health, findings from this review will be useful for researchers and communities who wish to collaborate to improve population health.

One of the main challenges that we anticipate for the realization of our scoping review is the time necessary to screen articles, as we expect that our search will yield a large number of studies. Discussions on the inclusion and exclusion criteria between the two reviewers and the research team prior to starting the screening process will contribute to ensuring our efficiency. We also recognize potential limitations of our scoping review. First, it is possible that we miss studies that could have been relevant to our scoping review objectives if they were published outside the scientific literature (*e.g.*, grey literature, reports from international or community organizations). Because we focus on articles written in English or French, we could also miss studies relevant to our objectives published in other languages. Our rigorous screening approach conducted by two independent reviewers will facilitate greater inter-reviewer reliability and maximize our chance of identifying all relevant studies. Second, the representation of the barriers and enablers of community participation as causal relationships through fuzzy cognitive

For peer review only

mapping is meant to illustrate soft models of causality needing empirical testing. The use of fuzzy cognitive mapping to synthesize the results from the scoping review however offers an operator-independent way to analyze and communicate the relative influence of the factors identified on community participation [28]. The literature-based map will in turn inform a mapping process involving stakeholders from Dhaka (Bangladesh), as part of the larger project.

The use of the *Weight of Evidence*, an innovative approach to knowledge synthesis whereby scientific and experiential knowledge are brought into conversation, will allow for the contextualization of the scoping review in the lived experience of stakeholders in Dhaka.[27,46,58] The procedures described in this scoping review protocol open the possibility for contextualizing literature reviews in lived experience in any context.

Better understanding the factors that influence the participation of communities in research could support a shift from researcher-driven health research towards research that is more inclusive of community voices and needs. Fostering authentic community participation in research can contribute to the movement for decolonizing public and global health. This can also bring benefits to marginalized communities through interventions that are more relevant to their contexts and needs.

ACKNOWLEDGEMENTS

The authors would like to thank the health librarians Sylvie Fontaine and Viviane Angers at the Université de Montréal for their support in developing this protocol. We would also like to thank members of the CIET/PRAM research lab at McGill University for sharing their expertise with us.

AUTHORS CONTRIBUTIONS

MCGD developed the scoping review protocol, the search strategy and wrote the first version of the manuscript. KZ and NA contributed to the development of the larger project to be conducted in Dhaka, as part of the COESA cluster randomized controlled trial. GF contributed to drafting the scoping review protocol. IS and NA provided expertise on fuzzy cognitive mapping, Harris' discourse analysis and the *Weight of Evidence* approach. All authors read, provided feedback, and approved the final manuscript.

FUNDING

This work was supported by the Fonds de recherche du Québec – Santé (FRQS) through a doctoral research scholarship awarded to the first author. This scoping review is part of a larger project supported by the Canadian Institutes of Health Research through the Project Grant program 201803PJT-400444-RC2-CFCA-120159. These institutions did not play a role in the development of this protocol.

COMPETING INTERESTS

None declared.

LEGENDS OF FIGURES

Figure 1. Example of a fuzzy cognitive map and associated concepts

Figure 2. Steps of the fuzzy cognitive mapping process for the scoping review. The icons represent the tools used in the different steps of the process (i.e., the PRISMA-ScR guidelines, Microsoft Excel, the software CIETmap 2.0). The illustrations below each step represent what each step will look like in practice.

For peer review only

REFERENCES

1. Winchester MS, BeLue R, Oni T, Wittwer-Backofen U, Deobagkar D, Onya H, et al. The Pan-University Network for Global Health: framework for collaboration and review of global health needs. Glob Health. 2016 Apr 21;12(1):13.
2. Elsey H, Agyepong I, Huque R, Quayyem Z, Baral S, Ebenso B, et al. Rethinking health systems in the context of urbanisation: challenges from four rapidly urbanising low-income and middle-income countries. BMJ Glob Health. 2019 Jun 1;4(3):e001501.
3. Friel S, Akerman M, Hancock T, Kumaresan J, Marmot M, Melin T, et al. Addressing the Social and Environmental Determinants of Urban Health Equity: Evidence for Action and a Research Agenda. J Urban Health. 2011 Aug 30;88(5):860.
4. Harpham T. Urban health in developing countries: What do we know and where do we go? Health Place. 2009 Mar 1;15(1):107–16.
5. Birn AE, Pillay Y, Holtz TH. Textbook of Global Health. Fourth edition. New York (NY): Oxford University Press; 2017. 674 p.
6. Affun-Adegbulu C, Adegbulu O. Decolonising Global (Public) Health: from Western universalism to Global pluriversalities. BMJ Glob Health. 2020 Aug 1;5(8):e002947.
7. Chaudhuri MM, Mkumba L, Raveendran Y, Smith RD. Decolonising global health: beyond 'reformative' roadmaps and towards decolonial thought. BMJ Glob Health. 2021 Jul 1;6(7):e006371.
8. Gautier L, Karambé Y, Dossou JP, Samb OM. Rethinking development interventions through the lens of decoloniality in sub-Saharan Africa: The case of global health. Glob Public Health. 2020 Dec 8;0(0):1–14.
9. Zarowsky C. Global health research, partnership, and equity: no more business-as-usual. BMC Int Health Hum Rights. 2011 Nov 8;11(2):S1.
10. Khan M, Abimbola S, Aloudat T, Capobianco E, Hawkes S, Rahman-Shepherd A. Decolonising global health in 2021: a roadmap to move from rhetoric to reform. BMJ Glob Health. 2021 Mar 1;6(3):e005604.
11. Jagosh J, Macaulay AC, Pluye P, Salsberg J, Bush PL, Henderson J, et al. Uncovering the Benefits of Participatory Research: Implications of a Realist Review for Health Research and Practice. Milbank Q. 2012 Jun;90(2):311–46.
12. Cargo M, Mercer SL. The Value and Challenges of Participatory Research: Strengthening Its Practice. Annu Rev Public Health. 2008 Apr 1;29(1):325–50.
13. Cyril S, Smith BJ, Possamai-Inesedy A, Renzaho AMN. Exploring the role of community engagement in improving the health of disadvantaged populations: a systematic review. [Review]. Glob Health Action. 2015;1:29842.
14. O'Mara-Eves A, Brunton G, McDaid D, Oliver S, Kavanagh J, Jamal F, et al. Community engagement to reduce inequalities in health: a systematic review, meta-analysis and economic analysis. Public Health Res. 2013 Nov;1(4):1–526.
15. World Population Review. World City Populations 2023 [Internet]. World Population Review. 2023 [cited 2023 Mar 17]. Available from: https://worldpopulationreview.com/world-cities
16. Ellis P, Roberts M. Leveraging Urbanization in South Asia: Managing Spatial Transformation for Prosperity and Livability [Internet]. Washington (DC): The World Bank; 2016 p. 207. Available from: https://openknowledge.worldbank.org/server/api/core/bitstreams/1002d994-c0ec-5654-a349-a5a18608314c/content

For peer review only - http://bmjopen.bmj.com/site/about/guidelines.xhtml

17. The World Bank Group. Population living in slums (% of urban population) - South Asia | Data [Internet]. The World Bank. 2023 [cited 2023 Mar 17]. Available from: https://data.worldbank.org/indicator/EN.POP.SLUM.UR.ZS?end=2020&locations=8S&most_recent_value_desc=true&start=2020&view=map&year=2020
18. de Snyder VNS, Friel S, Fotso JC, Khadr Z, Meresman S, Monge P, et al. Social Conditions and Urban Health Inequities: Realities, Challenges and Opportunities to Transform the Urban Landscape through Research and Action. J Urban Health. 2011 Dec 1;88(6):1183–93.
19. Mehrolhasani MH, Yazdi-Feyzabadi V, Ghasemi S. Community empowerment for health promotion in slums areas: A narrative review with emphasis on challenges and interventions. J Educ Health Promot. 2021 Jul 30;10:263.
20. Sheuya SA. Improving the Health and Lives of People Living in Slums. Ann N Y Acad Sci. 2008;1136(1):298–306.
21. Ahmed S, Meenar M. Just Sustainability in the Global South: A Case Study of the Megacity of Dhaka. J Dev Soc. 2018 Dec 1;34(4):401–24.
22. Adams AM, Rashid SF. Mobilising demand for primary health care services among urban slums: insights from a case study in Bangladesh. Dev Pract. 2021 Aug 18;31(6):726–38.
23. Vearey J, Luginaah I, Magitta NF, Shilla DJ, Oni T. Urban health in Africa: a critical global public health priority. BMC Public Health. 2019 Mar 25;19(1):340.
24. Grundy J, Wang X, Hirabayashi KC, Duncan R, Bersonda D, Eltayeb AO, et al. Health and immunisation services for the urban poor in selected countries of Asia. Infect Dis Poverty. 2019 Apr 18;8(1):26.
25. Otmani del Barrio M, Simard F, Caprara A. Supporting and strengthening research on urban health interventions for the prevention and control of vector-borne and other infectious diseases of poverty: scoping reviews and research gap analysis. Infect Dis Poverty. 2018 Sep 3;7(1):94.
26. Zinszer K, Caprara A, Lima A, Degroote S, Zahreddine M, Abreu K, et al. Sustainable, healthy cities: protocol of a mixed methods evaluation of a cluster randomized controlled trial for Aedes control in Brazil using a community mobilization approach. Trials. 2020 Feb 14;21(1):182.
27. Dion A, Carini-Gutierrez A, Jimenez V, Ben Ameur A, Robert E, Joseph L, et al. Weight of Evidence: Participatory Methods and Bayesian Updating to Contextualize Evidence Synthesis in Stakeholders' Knowledge. J Mix Methods Res [Internet]. 2021 Oct 13 [cited 2021 Nov 1]; Available from: https://doi.org/10.1177/15586898211037412
28. Sarmiento I, Paredes-Solís S, Dion A, Silver H, Vargas E, Cruz P, et al. Maternal health and Indigenous traditional midwives in southern Mexico: contextualisation of a scoping review. BMJ Open. 2021 Dec;11(12):e054542.
29. Peters M, Godfrey C, McInerney P, Munn Z, Trico A, Khalil H. Chapter 11: Scoping Reviews. In: Aromataris E, Munn Z, editors. JBI Manual for Evidence Synthesis [Internet]. JBI; 2020 [cited 2022 Feb 5]. Available from: https://wiki.jbi.global/display/MANUAL/Chapter+11%3A+Scoping+reviews
30. Peters MDJ, Godfrey C, McInerney P, Khalil H, Larsen P, Marnie C, et al. Best practice guidance and reporting items for the development of scoping review protocols. JBI Evid Synth. 2022 Apr;20(4):953–68.
31. Shamseer L, Moher D, Clarke M, Ghersi D, Liberati A, Petticrew M, et al. Preferred reporting items for systematic review and meta-analysis protocols (PRISMA-P) 2015:

For peer review only

elaboration and explanation. BMJ. 2015 Jan 2;349:g7647.
32. Tricco AC, Lillie E, Zarin W, O'Brien KK, Colquhoun H, Levac D, et al. PRISMA Extension for Scoping Reviews (PRISMA-ScR): Checklist and Explanation. Ann Intern Med. 2018 Oct 2;169(7):467–73.
33. Goodman RA, Bunnell R, Posner SF. What is "community health"? Examining the meaning of an evolving field in public health. Prev Med. 2014 Oct 1;67:S58–61.
34. MacQueen K, McLellan E, Metzger DS, Kegeles S, Strauss RP, Scotti R, et al. What Is Community? An Evidence-Based Definition for Participatory Public Health. Am J Public Health. 2001;91(12):1929–38.
35. World Health Organization. The 7th Global Conference on Health Promotion - Community Empowerment [Internet]. World Health Organization. 2009 [cited 2022 Aug 5]. Available from: https://www.who.int/teams/health-promotion/enhanced-wellbeing/seventh-global-conference/community-empowerment
36. Brunton G, Thomas J, O'Mara-Eves A, Jamal F, Oliver S, Kavanagh J. Narratives of community engagement: a systematic review-derived conceptual framework for public health interventions. [Review]. BMC Public Health. 2017 Dec;17(1):944.
37. Draper AK, Hewitt G, Rifkin S. Chasing the dragon: Developing indicators for the assessment of community participation in health programmes. Soc Sci Med. 2010 Sep;71(6):1102–9.
38. Key KD, Furr-Holden D, Lewis EY, Cunningham R, Zimmerman MA, Johnson-Lawrence V, et al. The Continuum of Community Engagement in Research: A Roadmap for Understanding and Assessing Progress. Prog Community Health Partnersh Res Educ Action. 2019;13(4):427–34.
39. Wallerstein N, Duran B. The theoretical, historical, and practice roots of CBPR. In: Community-Based Participatory Research for Health: Advancing Social and Health Equity. Third Edition. San Francisco (US): Jossey-Bass: A Wiley Brand; 2008. p. 17–30.
40. The World Bank. World Bank Country and Lending Groups [Internet]. The World Bank: Working for a World Free from Poverty. 2022 [cited 2022 Aug 25]. Available from: https://datahelpdesk.worldbank.org/knowledgebase/articles/906519-world-bank-country-and-lending-groups
41. The Cochrane Collaboration. LMIC Filters [Internet]. Cochrane Effective Practice and Organisation of Care: Trusted evidence, informed decisions, better health. 2022 [cited 2022 Aug 25]. Available from: https://epoc.cochrane.org/lmic-filters
42. Deuskar C. What does "urban" mean? [Internet]. World Bank Blogs. 2015 [cited 2022 Aug 25]. Available from: https://blogs.worldbank.org/sustainablecities/what-does-urban-mean
43. Morris M, Boruff JT, Gore GC. Scoping reviews: establishing the role of the librarian. J Med Libr Assoc. 2016;104(4):346–53.
44. Veritas Health Innovation. Covidence systematic review software [Internet]. Melbourne (Australia); 2022. Available from: www.covidence.org
45. Page A. Unnatural and Unequal: Social Determinants of Gender Inequality and Health and Their Impact on Disaster Management Interventions in Bangladesh [Internet] [Thesis]. Université d'Ottawa / University of Ottawa; 2015 [cited 2021 Jun 15]. Available from: http://ruor.uottawa.ca/handle/10393/33186
46. Sarmiento I, Cockcroft A, Dion A, Paredes-Solís S, De Jesús-García A, Melendez D, et al. Combining conceptual frameworks on maternal health in Indigenous communities - Fuzzy cognitive mapping using participant and operator-independent weighting. Field

For peer review only

Methods. 2022;34(3):223–39.
47. Andersson N, Silver H. Fuzzy cognitive mapping: An old tool with new uses in nursing research. J Adv Nurs. 2019 Dec;75(12):3823–30.
48. Gray S, Gray S, De Kok JL, Helfgott A, O'Dwyer B, Jordan R, et al. Using fuzzy cognitive mapping as a participatory approach to analyze change, preferred states, and perceived resilience of social-ecological systems. Ecol Soc [Internet]. 2015 [cited 2021 Nov 4];20(2). Available from: https://www.ecologyandsociety.org/vol20/iss2/art11/
49. Sarmiento I, Paredes-Solis S, Morris M, Pimentel J, Cockcroft A, Andersson N. Factors influencing maternal health in indigenous communities with presence of traditional midwifery in the Americas: protocol for a scoping review. BMJ Open. 2020;10(10):e037922.
50. Kosko B. Fuzzy cognitive maps. Int J Man-Mach Stud. 1986 Jan 1;24(1):65–75.
51. Kosko B. Hidden patterns in combined and adaptive knowledge networks. Int J Approx Reason. 1988 Oct 1;2(4):377–93.
52. Andersson N, Mitchell S. Epidemiological geomatics in evaluation of mine risk education in Afghanistan: introducing population weighted raster maps. Int J Health Geogr. 2006 Jan 3;5(1):1.
53. Sarmiento I, Paredes-Solis S, Loutfi D, Dion A, Cockcroft A, Andersson N. Fuzzy cognitive mapping and soft models of indigenous knowledge on maternal health in Guerrero, Mexico. BMC Med Res Methodol. 2020;20(1):125.
54. Niesink P, Poulin K, Šajna M. Computing transitive closure of bipolar weighted digraphs. Discrete Appl Math. 2013 Jan 1;161(1):217–43.
55. Harris ZS. Discourse Analysis. Language. 1952;28(1):1–30.
56. Arksey H, O'Malley L. Scoping studies: towards a methodological framework. Int J Soc Res Methodol. 2005 Feb 1;8(1):19–32.
57. Boyko JA, Lavis JN, Abelson J, Dobbins M, Carter N. Deliberative dialogues as a mechanism for knowledge translation and exchange in health systems decision-making. Soc Sci Med. 2012;75(11):1938–45.
58. Dion A, Nakajima A, McGee A, Andersson N. How Adolescent Mothers Interpret and Prioritize Evidence About Perinatal Child Protection Involvement: Participatory Contextualization of Published Evidence. Child Adolesc Soc Work J [Internet]. 2022 Jul 13 [cited 2022 Sep 21]; Available from: https://link.springer.com/10.1007/s10560-022-00865-1

For peer review only

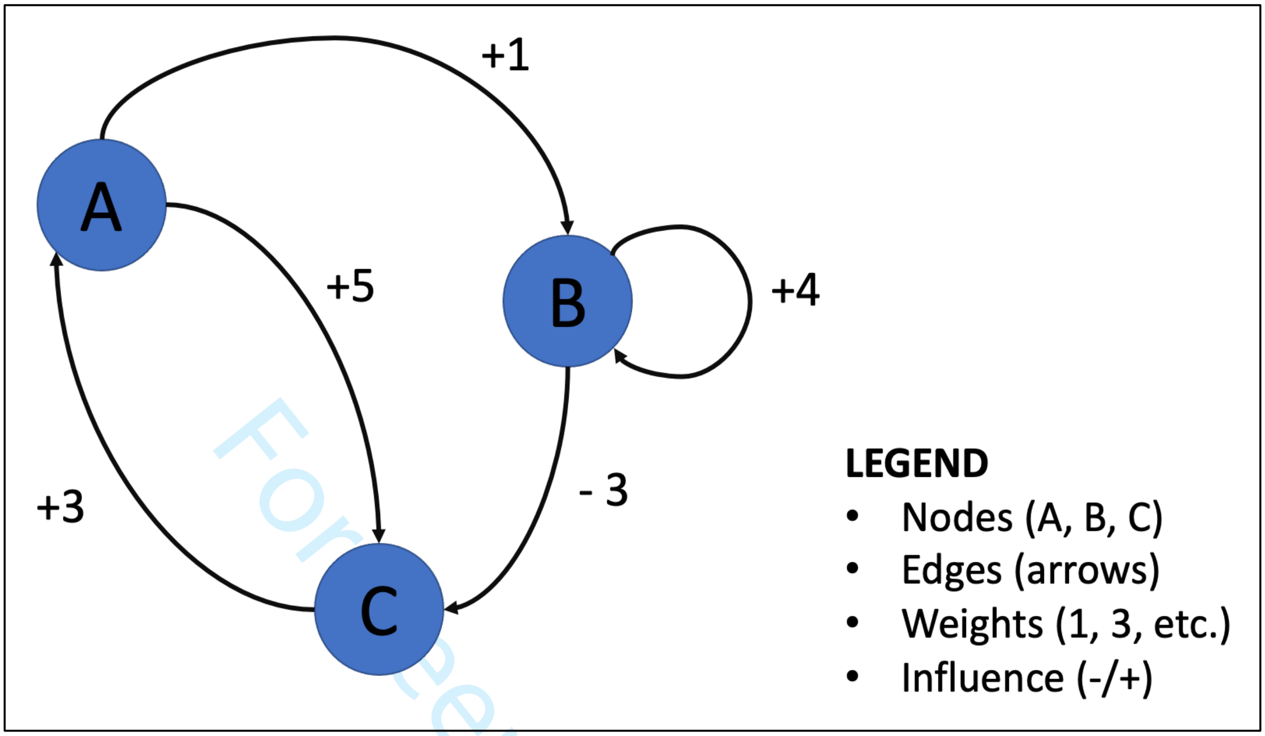

For peer review only

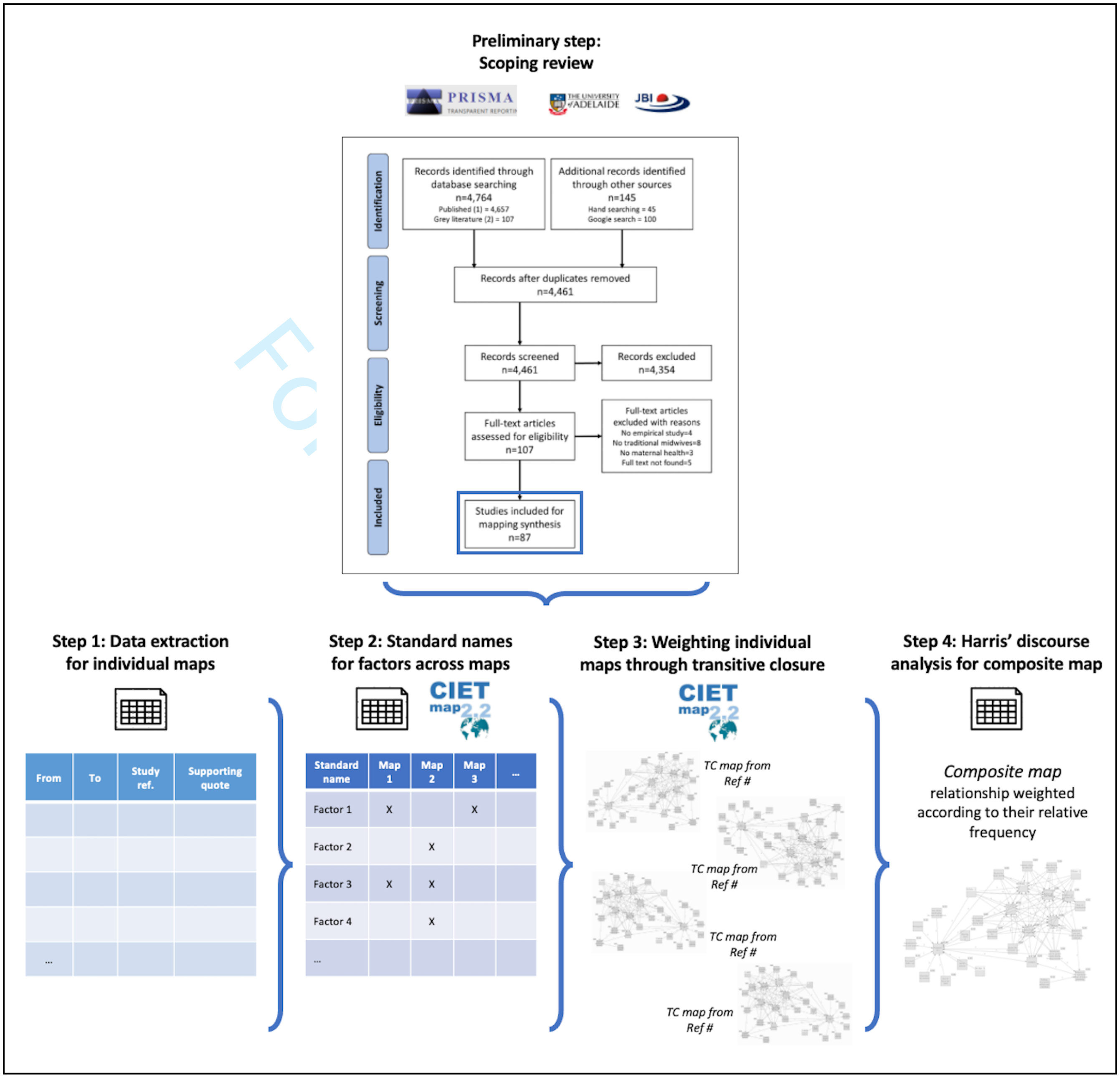

For peer review only - http://bmjopen.bmj.com/site/about/guidelines.xhtml

PRISMA-P 2015 Checklist

This checklist has been adapted for use with protocol submissions to *Systematic Reviews* from Table 3 in Moher D et al: Preferred reporting items for systematic review and meta-analysis protocols (PRISMA-P) 2015 statement. *Systematic Reviews* 2015 **4**:1

Section/topic	#	Checklist item	Information reported: Yes	Information reported: No	Line number(s)
**ADMINISTRATIVE INFORMATION**					
**Title**					
Identification	1a	Identify the report as a protocol of a systematic review	☒	☐	Page 1
Update	1b	If the protocol is for an update of a previous systematic review, identify as such	☐	☒	N/A
**Registration**	2	If registered, provide the name of the registry (e.g., PROSPERO) and registration number in the Abstract	☐	☒	N/A
**Authors**					
Contact	3a	Provide name, institutional affiliation, and e-mail address of all protocol authors; provide physical mailing address of corresponding author	☒	☐	Page 1
Contributions	3b	Describe contributions of protocol authors and identify the guarantor of the review	☒	☐	Page 12
**Amendments**	4	If the protocol represents an amendment of a previously completed or published protocol, identify as such and list changes; otherwise, state plan for documenting important protocol amendments	☐	☒	N/A
**Support**					
Sources	5a	Indicate sources of financial or other support for the review	☒	☐	Page 12
Sponsor	5b	Provide name for the review funder and/or sponsor	☒	☐	Page 12
Role of sponsor/funder	5c	Describe roles of funder(s), sponsor(s), and/or institution(s), if any, in developing the protocol	☒	☐	Page 12
**INTRODUCTION**					
**Rationale**	6	Describe the rationale for the review in the context of what is already known	☒	☐	Page 3 (bottom)
**Objectives**	7	Provide an explicit statement of the question(s) the review will address with reference to participants, interventions, comparators, and outcomes (PICO)	☒	☐	Page 4 (bottom)
**METHODS**					

BioMed Central
The Open Access Publisher

2

Section/topic	#	Checklist item	Information reported		Line number(s)
			Yes	No	
**Eligibility criteria**	8	Specify the study characteristics (e.g., PICO, study design, setting, time frame) and report characteristics (e.g., years considered, language, publication status) to be used as criteria for eligibility for the review	☒	☐	Pages 5-6
**Information sources**	9	Describe all intended information sources (e.g., electronic databases, contact with study authors, trial registers, or other grey literature sources) with planned dates of coverage	☒	☐	Page 5 (top); Page 6 (bottom)
**Search strategy**	10	Present draft of search strategy to be used for at least one electronic database, including planned limits, such that it could be repeated	☒	☐	Pages 6-8
STUDY RECORDS					
Data management	11a	Describe the mechanism(s) that will be used to manage records and data throughout the review	☒	☐	Page 8
Selection process	11b	State the process that will be used for selecting studies (e.g., two independent reviewers) through each phase of the review (i.e., screening, eligibility, and inclusion in meta-analysis)	☒	☐	Page 8
Data collection process	11c	Describe planned method of extracting data from reports (e.g., piloting forms, done independently, in duplicate), any processes for obtaining and confirming data from investigators	☒	☐	Pages 8-9
**Data items**	12	List and define all variables for which data will be sought (e.g., PICO items, funding sources), any pre-planned data assumptions and simplifications	☒	☐	Page 9 (point e)
**Outcomes and prioritization**	13	List and define all outcomes for which data will be sought, including prioritization of main and additional outcomes, with rationale	☒	☐	Pages 8-9 (points of data extraction form)
**Risk of bias in individual studies**	14	Describe anticipated methods for assessing risk of bias of individual studies, including whether this will be done at the outcome or study level, or both; state how this information will be used in data synthesis	☐	☒	N/A
DATA					
**Synthesis**	15a	Describe criteria under which study data will be quantitatively synthesized	☐	☒	N/A
	15b	If data are appropriate for quantitative synthesis, describe planned summary measures, methods of handling data, and methods of combining data from studies, including any planned exploration of consistency (e.g., I^2, Kendall's tau)	☐	☒	N/A
	15c	Describe any proposed additional analyses (e.g., sensitivity or subgroup analyses, meta-regression)	☐	☒	N/A
	15d	If quantitative synthesis is not appropriate, describe the type of summary planned	☒	☐	Pages 9-10
**Meta-bias(es)**	16	Specify any planned assessment of meta-bias(es) (e.g., publication bias across studies, selective reporting within studies)	☐	☒	N/A
**Confidence in cumulative evidence**	17	Describe how the strength of the body of evidence will be assessed (e.g., GRADE)	☐	☒	N/A

BioMed Central
The Open Access Publisher

For peer review only

3

For peer review only

BMJ Open

Why urban communities from low- and middle-income countries participate in public and global health research: Protocol for a scoping review

Journal:	*BMJ Open*
Manuscript ID	bmjopen-2022-069340.R2
Article Type:	Protocol
Date Submitted by the Author:	24-Apr-2023
Complete List of Authors:	Gagnon-Dufresne, Marie-Catherine; University of Montreal School of Public Health, Department of Social and Preventive Medicine; Centre de recherche en santé publique (CReSP)<br>Sarmiento, Ivan; McGill University, Department of Family Medicine; Universidad Del Rosario, Escuela de Medicina y Ciencias de la Salud<br>Fortin, Geneviève; University of Montreal, School of Public Health; Centre de recherche en santé publique (CReSP)<br>Andersson, Neil; McGill University, Department of Family Medicine; Universidad Autonoma de Guerrero - Campus Acapulco, Centro de Investigación de Enfermedades Tropicales (CIET)<br>Zinszer, Kate; University of Montreal School of Public Health, Department of Social and Preventive Medicine; Centre de recherche en santé publique (CReSP)
**Primary Subject Heading**:	Public health
Secondary Subject Heading:	Global health, Public health
Keywords:	PUBLIC HEALTH, PREVENTIVE MEDICINE, QUALITATIVE RESEARCH

SCHOLARONE™
Manuscripts

Why urban communities from low- and middle-income countries participate in public and global health research: Protocol for a scoping review

CORRESPONDING AUTHOR

Marie-Catherine Gagnon-Dufresne
École de santé publique, Université de Montréal
7101 Park Avenue, Montréal, QC
Canada H3N 1X9
marie-catherine.gagnon-dufresne@umontreal.ca

AUTHORS

Marie-Catherine Gagnon-Dufresne[1,2,3*], Iván Sarmiento[3,4], Geneviève Fortin[1,2], Neil Andersson[3,5], Kate Zinszer[1,2]

1. École de santé publique, Université de Montréal, Montréal (Canada)
2. Centre de recherche en santé publique (CReSP), Montréal (Canada)
3. CIET-PRAM, Department of Family Medicine, McGill University, Montréal (Canada)
4. Grupo de Estudios en Sistemas Tradicionales de Salud, Escuela de Medicina y Ciencias de la Salud, Universidad del Rosario, Bogotá (Colombia)
5. Centro de Investigación de Enfermedades Tropicales (CIET), Universidad Autónoma de Guerrero, Acapulco (Mexico)

WORD COUNT

3,971 words

For peer review only

Why urban communities from low- and middle-income countries participate in public and global health research: Protocol for a scoping review

ABSTRACT

Introduction: As the number of people living in cities increases worldwide, particularly in low- and middle-income countries (LMICs), urban health is a growing priority of public and global health. Rapid unplanned urbanization in LMICs has exacerbated inequalities, putting the urban poor at increasing risk of ill health due to difficult living conditions in cities. Collaboration with communities in research is a key strategy for addressing the challenges they face. The objective of this scoping review is therefore to identify factors that influence the participation of urban communities from LMICs in public and global health research.

Methods and analysis: We will develop a search strategy with a health librarian to explore the following databases: MEDLINE, Embase, Web of Science, Cochrane, Global Health, and CINAHL. We will use MeSH terms and keywords exploring the concepts of "low- and middle-income countries", "community participation in research", and "urban settings" to look at empirical research conducted in French or English. There will be no restriction in terms of dates of publication. Two independent reviewers will screen and select studies, first based on titles and abstracts, and then on full text. Two reviewers will extract data. We will summarize the results using tables and fuzzy cognitive mapping.

Ethics and dissemination: This scoping review is part of a larger project to be approved by the University of Montréal's Research Ethics Committee for Science and Health in Montréal (Canada), and the Institutional Review Board of the James P. Grant School of Public Health at BRAC University in Dhaka (Bangladesh). Results from the review will contribute to a participatory process seeking to combine scientific evidence with experiential knowledge of stakeholders in Dhaka to understand how to better collaborate with communities for research. The review could contribute to a shift towards research that is more inclusive and beneficial for communities.

KEYWORDS

public health, global health, community-based participatory research, stakeholder participation, urban population, urban health, fuzzy logic

STRENGTHS AND LIMITATIONS OF THIS STUDY

- What constitutes 'communities', 'participation in research' and 'urban settings' can be defined in various ways, so it will be crucial to highlight how these concepts are defined in the literature included in the scoping review.

For peer review only

For peer review only - http://bmjopen.bmj.com/site/about/guidelines.xhtml

- Reporting on community participation is heterogeneous, and identifying the research approaches, health issues, contexts, and community characteristics that favor participation will be challenging.
- The scoping review will summarize results using fuzzy cognitive mapping, providing soft models of causality that can be contextualized in the experience of local stakeholders in Dhaka.
- The methods presented in this scoping review protocol could be replicated to compare and combine scientific evidence and experiential knowledge anywhere.

INTRODUCTION

As the number of people living in cities increases worldwide, particularly in low- and middle-income countries (LMICs), the health of urban populations is a growing priority of public and global health.[1] Urbanization brings changes to the disease burdens, determinants of health, and patterns of health inequalities.[1,2] Despite the benefits of urban living and progress in population health, rapid and unplanned urbanization in LMICs has worsened health inequalities.[3] Not everyone in cities experiences these improvements equally, as policies and other efforts often fail to reach the most marginalized communities, including those living in informal settlements.[3] The urban poor are therefore at increased risk of ill health due to the difficult living conditions in cities.[3,4]

Several researchers in public and global health have criticized the reproduction of colonial relations in efforts to improve the health of populations in LMICs, as these efforts are often led by foreign researchers with little input from local populations.[5–7] The resulting unequal power dynamics between researchers and communities are among the reasons research makes little or mixed contributions to health.[8] In response, there are increasing calls to decolonize public and global health through community participation in research, to better meet their needs and ensure local relevance of the initiatives put in place to improve their health.[8–10]

Community participation in health research is recognized for building capacity and fostering conditions to enable better community control over determinants of their health.[11,12] Community participation can lead to equitable partnerships between communities and researchers, making research more empowering and effective.[12] However, most health research uses top-down community engagement approaches rather than bottom-up participatory methods.[13] While there is no standard definition of community participation, different uses of the term form a continuum from consulting or informing communities to sharing power with them.[13,14]

Some of the world's most populated cities are located in South Asian countries, including Bangladesh, India, and Pakistan.[15] These countries are characterized by high levels of urban poverty, with more than 50% of their urban population estimated to be living in informal settlements.[16,17] Considering that urbanization in these contexts is inextricably linked to complex patterns of discrimination and social exclusion for residents of informal

For peer review only - http://bmjopen.bmj.com/site/about/guidelines.xhtml

settlements, it is crucial that public/global health professionals and researchers collaborate with these communities to understand their health priorities and find innovative solutions to improve their health [18,19].

Collaborating with marginalized urban communities is a key strategy for addressing the many challenges they face.[3,20,21] Yet, these communities represent a particularly hard-to-reach group, as asymmetries in access to resources and opportunities affect their capacity to fully participate in and benefit from research implement to improve their health.[20,22] There is therefore an urgent need to better understand the barriers and enablers to their participation.[23–25]

The objective of this scoping review is to identify factors that influence the participation of urban communities from low- and middle-income countries in public and global health research. Part of a larger project, this scoping review will contribute to a dialogue between scientific and experiential knowledge on the factors that influence community participation in public and global health research. We will contextualize the results from the scoping review in the views of stakeholders in Dhaka (Bangladesh) in a participatory process to reflect their experiences. This contextualization will identify barriers and enablers to participation that are specific to Dhaka, in preparation for a cluster randomized controlled trial testing the effect of a participatory community mobilization intervention for reducing dengue infection.[26]

METHODS AND ANALYSIS

This review is part of a larger project aimed at comparing and combining different knowledge sources to provide a knowledge base for decision making, that will be used to inform a cluster randomized controlled trial to reduce dengue infection in Dhaka.[26] The larger project will consider four knowledge sources: 1) the scoping review described in this protocol; 2) the views of Canadian and Bangladeshi public and global health researchers; 3) the views of personnel from community-based organizations in Dhaka; 4) the views of community members from underserved neighbourhoods in Dhaka. We will adapt the *Weight of Evidence* approach [27,28] and use fuzzy cognitive mapping to bring these different knowledge sources into conversation.

In this protocol, we focus on describing the procedures to conduct the scoping review and briefly discuss how the results will be used to inform the subsequent phases of the larger project. The proposed scoping review will be conducted in accordance with the PRISMA-ScR (Preferred Reporting Items for Systematic Reviews and Meta-Analyses – Extension for Scoping Reviews) guidelines and Joanna Briggs Institute's methodology to ensure accuracy, completeness and transparency.[29–32]

Review questions

This scoping review will seek to answer the following question, developed according to the PCC method (Participants, Concept, Context) recommended by the Joanna Briggs Institute [29]: What factors influence the participation of urban communities from low- and middle-income countries in research, based on evidence from the public and global health literature?

For peer review only

For peer review only - http://bmjopen.bmj.com/site/about/guidelines.xhtml

The review will also seek to answer the following sub-questions:

1) What are the main barriers and enablers of community participation in public and global health research?
2) What is the relative influence of these factors on community participation?
3) What research approaches are most and least favourable to community participation?
4) What public and global health issues are most and least favourable to community participation?
5) What contexts are most and least favourable to community participation?
6) What community characteristics are most and least favourable to community participation?

Table 1 summarizes the eligibility criteria for the scoping review following the PCC (Participants, Concept, Context) method. These criteria will be explained in more detail in the next section.

Table 1. Eligibility criteria for the scoping review, based on the PCC method

PCC element	Correspondence in the scoping review
Participants	Communities in urban settings:<br>• Communities are defined as groups of individuals linked by shared social ties or interests who engage in joint actions.[33–35]<br>• Urban settings, as defined by the authors of the articles included in the scoping review.
Concept	Community participation in research<br>• Research involving non-academic stakeholder in decision-making over some aspect of the research.[14]
Context	Low- and middle-income countries<br>• Countries included in the World Bank's classification of low- and middle-income countries based on gross national incomes per capita.[36]

Eligibility criteria

Types of sources

This scoping review will consider empirical studies with quantitative, qualitative, and mixed methods designs. We will not include literature reviews and meta-analyses, but we will consider including the empirical studies reported in reviews and meta-analyses if relevant. We will not consider grey or scientific literature reporting on programs, policy or other initiative implemented outside of research purposes, since our focus is community participation in *research.*

Participants

Participants for this review will be communities in urban settings. Because this review will look at research participation at the community level rather than at the individual level, we will exclude studies discussing the participation of individuals in research (*i.e.*, patient engagement, individual motivation to participate). The term 'community' generally refers to population groups and the locus (*i.e.*, place, venue, or other units) of their actions.[33]

For peer review only - http://bmjopen.bmj.com/site/about/guidelines.xhtml

For this scoping review, we define communities as groups of people with diverse characteristics that are linked by social ties or identities; share common interests or concerns; and engage in joint action in settings, venues or areas that may be physically, geographically, culturally or politically defined.[33–35] The definition of what constitutes a community will therefore remain broad for this scoping review to ensure that we consider all relevant studies discussing the participation of communities in public and global health research.

We will however focus on communities located in urban settings, excluding rural communities. There is no standard international definition of what constitutes an urban setting. Each country has its own definition, following nationally defined criteria on population size, population density, type of economic activity, physical characteristics, level of infrastructure, or other characteristics.[37] Considering the lack of a common definition, the scoping review will consider all studies in LMICs conducted in urban settings or cities as identified by the authors, including neighbourhoods and informal settlements (slums) in cities.

Our focus on urban communities in low- and middle-income countries is justified by the fact that the larger project is part of a cluster randomized controlled trial on dengue which will be conducted in Dhaka (Bangladesh), and that the factors influencing community participation in research will likely vary between rural and urban communities, and between high- and low- and middle-income countries.

Concept

Community-engaged research is a broad topic, defined in various ways and used for various reasons. It is often an umbrella term for research involving the participation of non-academic stakeholders, with diverse models and conceptual frameworks.[14] There is no standard definition of community participation in research in public and global health.[13,14] The distinction between 'engagement,' 'mobilization' and 'participation' in research is unclear, as these terms are often used interchangeably and with changing definitions.

Various authors discuss the levels of community participation in research as being positioned along a continuum, ranging from information provision and exchange, to consultation, to co-production, and to shared leadership and community control.[14,38–41] For this scoping review, we will not restrict the search to a specific level of participation, but we will examine and compare how different approaches (i.e., community mobilization interventions, partnered research, community-based participatory research designs, etc.) are found to enable or hinder participation. However, research in which there is little community involvement (*i.e.*, health education, consultation efforts in which communities have no decision-making power over some aspects of the research) will be excluded.

Context

This review will focus on low- and middle-income countries. The definition of low- and middle-income countries used for the review is based on the World Bank's classification from the 2023 fiscal year, established following a country's gross national incomes per

For peer review only - http://bmjopen.bmj.com/site/about/guidelines.xhtml

capita.[36] The Cochrane Effective Practice and Organisation of Care (EPOC) group has developed a filter for literature reviews based on the World Bank classification to identify studies relevant to LMICs.[42]

Exclusion criteria

To ensure the selection of relevant studies for the review, we will use the following exclusion criteria:

- Grey literature (institutional reports from NGOs, policy documents or other document not reporting on research projects)
- Not empirical research
- Discussing community engagement, participation, partnership, or mobilization in contexts other than research (*i.e.*, programs, policy, urban planning)
- Reports on individual engagement in research (*i.e.*, patient engagement, individual motivation) or on the individual experiences of participants
- Not discussing factors that influence community participation in research
- Conducted in contexts other than urban settings
- Conducted in countries other than low- and middle-income countries
- Full text of the reference not available

Table 2 summarizes the inclusion and exclusion criteria used to select articles for the scoping review.

Table 2. Inclusion and exclusion criteria for the scoping review

Inclusion criteria	Exclusion criteria
• Empirical qualitative, quantitative, and mixed methods research<br>• Discussing community engagement, participation, partnership or mobilization in research<br>• Focused on community-level engagement<br>• Discussing factors that influence community participation in research<br>• Conducted in urban settings<br>• Conducted in low- and middle-income countries	• Not empirical research<br>• Grey literature, including reports from NGOs or policy documents<br>• Discussing community engagement, participation, partnership, or mobilization in contexts other than research<br>• Focused on individual-level engagement or on individual experiences of participants<br>• Not discussing factors that influence community participation in research<br>• Conducted in contexts other than urban settings<br>• Conducted in countries other than low- and middle-income countries<br>• Full text of the reference not available

Search strategy

The search strategy will be developed with the help of a health librarian. It will explore the following databases: MEDLINE, Embase, Web of Science, Cochrane, Global Health, and CINAHL. We will use MeSH terms and keywords to identify studies reported in English or French. We will not have restrictions in terms of dates of publication. We will not contact the authors of the articles selected to request additional information. Table 3 presents the initial search strategy for MEDLINE, which will be adapted for each database.

For peer review only - http://bmjopen.bmj.com/site/about/guidelines.xhtml

Table 3. Example of a potential search strategy for MEDLINE

**Concept 1: Low- and middle-income countries**
1. (afghanistan or albania or algeria or american samoa or angola or "antigua and barbuda" or antigua or barbuda or argentina or armenia or armenian or aruba or azerbaijan or bahrain or bangladesh or barbados or "republic of Belarus" or belarus or byelarus or belorussia or byelorussian or belize or british honduras or benin or dahomey or bhutan or bolivia or "bosnia and herzegovina" or bosnia or herzegovina or botswana or bechuanaland or brazil or brasil or bulgaria or burkina faso or burkina fasso or upper volta or burundi or urundi or cabo verde or cape verde or cambodia or kampuchea or khmer republic or cameroon or cameron or cameroun or central african republic or ubangi shari or chad or chile or china or colombia or comoros or comoro islands or iles comores or mayotte or "democratic republic of the congo" or democratic republic congo or congo or zaire or costa rica or "cote d'ivoire" or "cote d' ivoire" or cote divoire or cote d ivoire or ivory coast or croatia or cuba or cyprus or czech republic or czechoslovakia or djibouti or french somaliland or dominica or dominican republic or ecuador or egypt or united arab republic or el salvador or equatorial guinea or spanish guinea or eritrea or estonia or eswatini or swaziland or ethiopia or fiji or gabon or gabonese republic or gambia or "georgia (republic)" or georgian or ghana or gold coast or gibraltar or greece or grenada or guam or guatemala or guinea or guinea bissau or guyana or british guiana or haiti or hispaniola or honduras or hungary or india or indonesia or timor or iran or iraq or "isle of man" or jamaica or jordan or kazakhstan or kazakh or kenya or "democratic people's republic of korea" or "republic of korea" or north korea or south korea or korea or kosovo or kyrgyzstan or kirghizia or kirgizstan or kyrgyz republic or kirghiz or laos or lao pdr or "lao people's democratic republic" or latvia or lebanon or lebanese republic or lesotho or basutoland or liberia or libya or libyan arab jamahiriya or lithuania or macau or macao or "republic of north macedonia" or macedonia or madagascar or malagasy republic or malawi or nyasaland or malaysia or malay federation or malaya federation or maldives or indian ocean islands or indian ocean or mali or malta or micronesia or "federated states of Micronesia" or kiribati or marshall islands or nauru or northern mariana islands or palau or tuvalu or mauritania or mauritius or mexico or moldova or moldovian or mongolia or montenegro or morocco or ifni or mozambique or portuguese east africa or myanmar or burma or namibia or nepal or netherlands antilles or nicaragua or niger or nigeria or oman or muscat or pakistan or panama or papua new guinea or new guinea or paraguay or peru or philippines or philipines or phillipines or phillippines or poland or "polish people's republic" or portugal or portuguese republic or puerto rico or romania or russia or russian federation or ussr or soviet union or "union of soviet socialist republics" or rwanda or ruanda or samoa or pacific islands or polynesia or samoan islands or navigator island or navigator islands or "sao tome and principe" or saudi arabia or senegal or serbia or seychelles or sierra leone or slovakia or slovak republic or slovenia or melanesia or solomon island or solomon islands or norfolk island or norfolk islands or somalia or south africa or south sudan or sri lanka or ceylon or "saint kitts and nevis" or "st. kitts and nevis" or saint lucia or "st. lucia" or "saint vincent and the grenadines" or saint vincent or "st. vincent" or grenadines or sudan or suriname or surinam or dutch guiana or netherlands guiana or syria or syrian arab republic or tajikistan or tadjikistan or tadzhikistan or tadzhik or tanzania or tanganyika or thailand or siam or timor leste or east timor or togo or togolese republic or tonga or "trinidad and tobago" or trinidad or tobago or tunisia or turkey or turkmenistan or turkmen or uganda or ukraine or uruguay or uzbekistan or uzbek or vanuatu or new hebrides or venezuela or vietnam or viet nam or middle east or west bank or gaza or palestine or yemen or yugoslavia or zambia or zimbabwe or northern rhodesia or global south or "africa south of the sahara" or sub-saharan africa or subsaharan africa or africa, central or central africa or africa, northern or north africa or northern africa or magreb or maghrib or sahara or africa, southern or southern africa or africa, eastern or east africa or eastern africa or africa, western or west africa or western africa or west indies or indian ocean islands or caribbean or central america or latin america or "south and central america" or south america or asia, central or central asia or asia, northern or north asia or northern asia or asia, southeastern or southeastern asia or south eastern asia or southeast asia or south east asia or asia, western or western asia or europe, eastern or east europe or eastern europe or developing country or developing countries or developing nation? or developing population? or developing world or less developed countr* or less developed nation? or less developed population? or less developed world or lesser developed countr* or lesser developed nation? or lesser developed population? or lesser developed world or under developed countr* or under developed nation? or under developed population? or under developed world or underdeveloped countr* or underdeveloped nation?

For peer review only - http://bmjopen.bmj.com/site/about/guidelines.xhtml

or underdeveloped population? or underdeveloped world or middle income countr* or middle income nation? or middle income population? or low income countr* or low income nation? or low income population? or lower income countr* or lower income nation? or lower income population? or underserved countr* or underserved nation? or underserved population? or underserved world or under served countr* or under served nation? or under served population? or under served world or deprived countr* or deprived nation? or deprived population? or deprived world or poor countr* or poor nation? or poor population? or poor world or poorer countr* or poorer nation? or poorer population? or poorer world or developing econom* or less developed econom* or lesser developed econom* or under developed econom* or underdeveloped econom* or middle income econom* or low income econom* or lower income econom* or low gdp or low gnp or low gross domestic or low gross national or lower gdp or lower gnp or lower gross domestic or lower gross national or lmic or lmics or third world or lami countr* or transitional countr* or emerging economies or emerging nation?).ti,ab,sh,kf.<br>2. Developing countries/<br>3. 1 or 2
**Concept 2: Community participation in research**
4. (((participat* or communit* or partner*) adj3 research) or (communit* adj3 (participat* or engage* or mobili?ation or intervention*)) or participatory or CBPR).ti,ab,sh,kf.<br>5. Community-based participatory research/<br>6. Community participation/<br>7. 4 or 5 or 6
**Concept 3: Urban settings**
8. (urban* or city or cities or metropol* or megacit* or megalop* or municipalit* or "informal settlement" or "informal settlements" or slum* or favela* or "shanty town" or "shanty towns" or ghetto* or bustee*).ti,ab,sh,kf.<br>9. Urban Health/<br>10. Urban Population/<br>11. Cities/<br>12. Urbanization/<br>13. Poverty Areas/<br>14. 8 or 9 or 10 or 11 or 12 or 13
**Final search strategy**
15. 3 and 7 and 14

The search strategy will be developed with the input from a librarian and the research team to identify new keywords.[43] After our initial screening in MEDLINE, we will search the included articles for new keywords. A new search will then be conducted combining the newly found MeSH terms and keywords to the existing search. A librarian will assess whether these new terms should be included in the final search strategy. When all articles are screened, we will search the reference lists of selected studies to identify additional studies meeting our inclusion criteria.

Study selection

Following the search, all identified citations will be collated and uploaded into Covidence,[44] and duplicates will be removed. Study selection will be conducted in two phases by two independent reviewers, who will reconcile differences by consensus. A third independent reviewer will help resolve any further disagreement.

The initial screening of the retrieved sources will use titles and abstracts. The second phase of selection will use full text. Reasons for excluding sources at full text that do not meet the inclusion criteria will be recorded and reported in the review. The results of the

For peer review only

search and the study selection process will be reported in the flow diagram developed by PRISMA-ScR.[32,45]

Because the aim of scoping reviews is to map the available evidence on a specific topic, we will not perform an assessment of the methodological quality or risk of bias of the articles included in the review.[29] However, the data extraction form will report the research design as well as the data collection and analysis methods of selected articles. This will allow us to dress a portrait of the available evidence on the factors influencing the participation of urban communities in research.

Data extraction

Two reviewers will develop and pilot a data extraction form, and extract the data in Covidence.[44] The form will include:

a. Details on the study (title, names of the authors, year of publication, study objectives, research design, and data collection and analysis methods)
b. The country and the urban settings in which studies were conducted
c. Characteristics of participating communities
d. If available, the definitions of 'community' and 'urban setting' used by the authors
e. The participation approach used and the extent of community participation
f. The findings regarding the factors (barriers, enablers, and other factors) influencing the participation of urban communities in public and global health research
g. If available, the relative influence (qualitative or quantitative) of the factors identified on community participation
h. If available, other relations among the factors identified, and their relative influence on community participation
i. Explanation of the relationships between factors (quotes from the articles)

We will not systematically extract data on the results of the studies since this is outside the scope of the review objectives and research question.

The data extraction form will be piloted before beginning the study selection process with a random sample of 5 studies among all the studies to be reviewed. The pilot test will help identify missing data and will contribute to ensuring that the reporting of participation approaches and factors influencing community participation is coherent across studies and between the two reviewers. The data extraction form will be modified and revised as necessary, in an iterative manner, during the data extraction process. Modifications will be detailed in the report of the review.

Any disagreements on data extraction that arise between the two reviewers at the pilot or data extraction stages will be resolved by consensus, or by discussion with a third independent reviewer if necessary.

Data analysis and presentation

The presentation of results will follow the PRISMA-ScR guidelines.[32] We will present the results in tables and use fuzzy cognitive mapping (FCM) to illustrate how the different

For peer review only - http://bmjopen.bmj.com/site/about/guidelines.xhtml

factors identified influence community participation in research, adapting the *Weight of Evidence* approach.[27,46] A narrative summary will also accompany the tabulated and mapped results, describing how the results relate to the review objectives and questions.

FCM uses graph theory and fuzzy logic to generate soft models of how change could happen based on assumed causal relationships.[47–49] These soft models are illustrated through graphs called fuzzy cognitive maps (Figure 1), which are used to represent assumed causal relationships between concepts.[50,47] The maps use nodes (factors affecting the issue) and edges (arrows representing the relationships between factors), weighted by the relative strength of their influence on the issue of interest.[51,47,27] Depending on the knowledge source of the maps, edges can have different values (hence the term *fuzzy*) to quantify their influence in a relative way.[49]

FCM will be the cornerstone for the presentation of the scoping review, through the creation of fuzzy cognitive maps to represent: 1) each article included in the review; and 2) a composite map for the whole review. FCM will allow to summarize in a composite map the relative influence that each factor might have on community participation, in relation to all the other factors identified in the review.[27,28,47] We will go through several steps (detailed below) to create the composite literature-based fuzzy cognitive map of the barriers and enablers to community participation (Figure 2).

First, we will create one fuzzy cognitive map for each article selected in the scoping review (Step 1 in Figure 2). In each individual map, community participation will be the outcome of interest. We will include each barrier and enabler of community participation mentioned in the article (point e in the data extraction form) as a node in the map, which we will organize in a table. This table will have two initial columns indicating the origin factor (from) and the consequence factor (to). Additional columns will present the evidence supporting the relationship between both factors from the article (point h in the data extraction form). Each relationship identified will be a row in the table.[49]

Second, once all the individual tables are created, we will standardize the names of the factors across the individual articles so that they can be comparable (Step 2 in Figure 2).[46] On each individual map reporting the relationships identified in each study, we will calculate fuzzy transitive closure in the open access CIETmap 2.0 (Step 3 in Figure 2).[52] Fuzzy transitive closure is a mathematical model used to calculate the influence of each relationship on community participation, considering all the possible relationships represented in the map.[53,54] After transitive closure, each relationship will have a value between 0 (having no influence) and 1 (having the strongest influence) to represent the relative strength of their influence on community participation, with positive and negative signs indicating whether the relationship is stimulative or inhibitive.[27,53]

Third, we will create a composite map for the whole review (Step 4 in Figure 2). To create this composite map, we will attribute weights to each node using Harris' discourse analysis, an analytical approach developed in the 1950s based the frequency of occurrence of discourse elements sharing similar meanings in a body of text, such as a literature review.[55,46] We will consider the frequency of occurrence of each relationship across the maps developed for each article in the scoping review. This means that a factor

For peer review only - http://bmjopen.bmj.com/site/about/guidelines.xhtml

that is repeated in multiple maps would have a stronger causal meaning for community participation than a factor only mentioned in one or two maps.[46] We will establish the relative frequency of factors by dividing each occurrence by the highest frequency across all the maps. We will therefore obtain a value between 0 for the relationships that did not exist and 1 for the relationship most frequently mentioned.[46] These different steps will allow us to create a composite map representing all the factors identified in the scoping review, weighted according to their relative frequency.

Patient and public involvement

We will include a consultation phase in the scoping review, as Arksey & O'Malley (2005) recognize the benefit of discussing the results of a review with experts.[56] The *Weight of Evidence* approach, which we will adapt for this scoping review, advocates for experiential knowledge to be considered on an equal footing with the evidence synthesized from the literature.[27] Therefore, in the context of the larger project, which adopts a participatory methodology and involves a community advisory board, people concerned with the issue of interest (*i.e.*, participation of urban communities in health research) will be invited to contextualize the scoping review.

After conducting the scoping review, stakeholders in Dhaka will develop their own fuzzy cognitive maps on the factors that they believe can influence community participation in the Bangladeshi context. After developing their maps, they will interpret the literature-based map from the scoping review by comparing the results with their own maps. We will seek the perspectives of three stakeholder groups, namely public and global health researchers, community-based organizations, and community stakeholders.

Finally, we will use the composite map from the scoping review and the various maps from these three stakeholder groups to generate a final map incorporating these two knowledge sources. The literature-based map, the stakeholder maps and this final map will be reviewed through deliberative dialogue with stakeholders in Dhaka.[57] The maps and discussions with stakeholders will inform decision-making for the cluster randomized controlled trial on dengue testing a participatory community mobilization intervention, where communities in Dhaka will develop their own solutions to reduce dengue infection. These steps will be conducted and reported separately.

ETHICS AND DISSEMINATION

This scoping review does not require ethics approval. However, the consultation process is part of a larger project which will need to be approved by the University of Montréal's Research Ethics Committee for Science and Health in Montréal (Canada), and the Institutional Review Board of the James P. Grant School of Public Health at BRAC University in Dhaka (Bangladesh). We will apply for ethics approval for the larger project at both universities by August 2023. We will share the results from the scoping review with the scientific community through scientific articles and presentations at conferences, and with local stakeholders in Dhaka through a participatory process involving fuzzy cognitive mapping and deliberative dialogue. Results from this process will directly inform the implementation of the cluster randomized controlled trial on dengue in Dhaka.[26]

For peer review only

For peer review only - http://bmjopen.bmj.com/site/about/guidelines.xhtml

DISCUSSION

This protocol described a scoping review which will seek to identify and map the factors that can influence the participation of urban communities from low- and middle-income countries in public and global health research. The review will contribute to the understanding of how to foster the participation of these communities in research, so that it can better respond to local needs. Given that marginalized urban communities represent a particularly hard-to-reach group in research and that urban health is a growing priority of public and global health, findings from this review will be useful for researchers and communities who wish to collaborate to improve population health.

The use of the *Weight of Evidence*, an innovative approach to knowledge synthesis whereby scientific and experiential knowledge are brought into conversation, will allow for the contextualization of the scoping review in the lived experience of stakeholders in Dhaka.[27,46,58] The procedures described in this scoping review protocol open the possibility for contextualizing literature reviews in lived experience in any context.

One of the main challenges that we anticipate for the realization of our scoping review is the time necessary to screen articles, as we expect that our search will yield a large number of studies. Discussions on the inclusion and exclusion criteria between the two reviewers and the research team prior to starting the screening process will contribute to ensuring our efficiency. We also recognize potential limitations of our scoping review. First, it is possible that we miss studies that could have been relevant to our scoping review objectives if they were published outside the scientific literature (*e.g.*, grey literature, reports from international or community organizations). Because we focus on articles written in English or French, we could also miss studies relevant to our objectives published in other languages. Our rigorous screening approach conducted by two independent reviewers will facilitate greater inter-reviewer reliability and maximize our chance of identifying all relevant studies. Second, the representation of the barriers and enablers of community participation as causal relationships through fuzzy cognitive mapping is not meant to illustrate probability, but rather to represent soft models of causality that need empirical testing. In addition, our identification and classification of barriers and enablers of community participation rest on our subjective interpretation of the evidence. However, the use of fuzzy cognitive mapping and Harris' discourse analysis to synthesize the results from the scoping review offers an operator-independent way to analyze and communicate the relative influence of the factors identified on community participation.[28] The literature-based map will in turn inform a mapping process involving stakeholders from Dhaka (Bangladesh), as part of the larger project. Third, we recognize that most research conducted in urban settings in LMICs focus on urban poor populations. It is therefore possible that most of the studies included in our review discuss underserved or marginalized populations, which is not necessarily representative of all communities living in cities in LMICs.

Better understanding the factors that influence the participation of communities in research could support a shift from researcher-driven health research towards research that is more inclusive of community voices and needs. Fostering authentic community

For peer review only - http://bmjopen.bmj.com/site/about/guidelines.xhtml

participation in research can contribute to the movement for decolonizing public and global health. This can also bring benefits to marginalized communities through interventions that are more relevant to their contexts and needs.

ACKNOWLEDGEMENTS

The authors would like to thank the health librarians Sylvie Fontaine and Viviane Angers at the Université de Montréal for their support in developing this protocol. We would also like to thank members of the CIET/PRAM research lab at McGill University for sharing their expertise with us.

AUTHORS CONTRIBUTIONS

MCGD developed the scoping review protocol, the search strategy and wrote the first version of the manuscript. KZ and NA contributed to the development of the larger project to be conducted in Dhaka, as part of the COESA cluster randomized controlled trial. GF contributed to drafting the scoping review protocol. IS and NA provided expertise on fuzzy cognitive mapping, Harris' discourse analysis and the *Weight of Evidence* approach. All authors read, provided feedback, and approved the final manuscript.

DATA AVAILABILITY

Not applicable

FUNDING

This work was supported by the Fonds de recherche du Québec – Santé (FRQS) through a doctoral research scholarship awarded to the first author. This scoping review is part of a larger project supported by the Canadian Institutes of Health Research through the Project Grant program 201803PJT-400444-RC2-CFCA-120159. These institutions did not play a role in the development of this protocol.

COMPETING INTERESTS

None declared.

LEGENDS OF FIGURES

Figure 1. Example of a fuzzy cognitive map and associated concepts

Figure 2. Steps of the fuzzy cognitive mapping process for the scoping review.

For peer review only

REFERENCES

1. Winchester MS, BeLue R, Oni T, Wittwer-Backofen U, Deobagkar D, Onya H, et al. The Pan-University Network for Global Health: framework for collaboration and review of global health needs. Glob Health. 2016 Apr 21;12(1):13.
2. Elsey H, Agyepong I, Huque R, Quayyem Z, Baral S, Ebenso B, et al. Rethinking health systems in the context of urbanisation: challenges from four rapidly urbanising low-income and middle-income countries. BMJ Glob Health. 2019 Jun 1;4(3):e001501.
3. Friel S, Akerman M, Hancock T, Kumaresan J, Marmot M, Melin T, et al. Addressing the Social and Environmental Determinants of Urban Health Equity: Evidence for Action and a Research Agenda. J Urban Health. 2011 Aug 30;88(5):860.
4. Harpham T. Urban health in developing countries: What do we know and where do we go? Health Place. 2009 Mar 1;15(1):107–16.
5. Birn AE, Pillay Y, Holtz TH. Textbook of Global Health. Fourth edition. New York (NY): Oxford University Press; 2017. 674 p.
6. Affun-Adegbulu C, Adegbulu O. Decolonising Global (Public) Health: from Western universalism to Global pluriversalities. BMJ Glob Health. 2020 Aug 1;5(8):e002947.
7. Chaudhuri MM, Mkumba L, Raveendran Y, Smith RD. Decolonising global health: beyond 'reformative' roadmaps and towards decolonial thought. BMJ Glob Health. 2021 Jul 1;6(7):e006371.
8. Gautier L, Karambé Y, Dossou JP, Samb OM. Rethinking development interventions through the lens of decoloniality in sub-Saharan Africa: The case of global health. Glob Public Health. 2020 Dec 8;0(0):1–14.
9. Zarowsky C. Global health research, partnership, and equity: no more business-as-usual. BMC Int Health Hum Rights. 2011 Nov 8;11(2):S1.
10. Khan M, Abimbola S, Aloudat T, Capobianco E, Hawkes S, Rahman-Shepherd A. Decolonising global health in 2021: a roadmap to move from rhetoric to reform. BMJ Glob Health. 2021 Mar 1;6(3):e005604.
11. Jagosh J, Macaulay AC, Pluye P, Salsberg J, Bush PL, Henderson J, et al. Uncovering the Benefits of Participatory Research: Implications of a Realist Review for Health Research and Practice. Milbank Q. 2012 Jun;90(2):311–46.
12. Cargo M, Mercer SL. The Value and Challenges of Participatory Research: Strengthening Its Practice. Annu Rev Public Health. 2008 Apr 1;29(1):325–50.
13. Cyril S, Smith BJ, Possamai-Inesedy A, Renzaho AMN. Exploring the role of community engagement in improving the health of disadvantaged populations: a systematic review. [Review]. Glob Health Action. 2015;1:29842.
14. O'Mara-Eves A, Brunton G, McDaid D, Oliver S, Kavanagh J, Jamal F, et al. Community engagement to reduce inequalities in health: a systematic review, meta-analysis and economic analysis. Public Health Res. 2013 Nov;1(4):1–526.
15. World Population Review. World City Populations 2023 [Internet]. World Population Review. 2023 [cited 2023 Mar 17]. Available from: https://worldpopulationreview.com/world-cities
16. Ellis P, Roberts M. Leveraging Urbanization in South Asia: Managing Spatial Transformation for Prosperity and Livability [Internet]. Washington (DC): The World Bank; 2016 p. 207. Available from: https://openknowledge.worldbank.org/server/api/core/bitstreams/1002d994-c0ec-5654-a349-a5a18608314c/content

For peer review only - http://bmjopen.bmj.com/site/about/guidelines.xhtml

17. The World Bank Group. Population living in slums (% of urban population) - South Asia | Data [Internet]. The World Bank. 2023 [cited 2023 Mar 17]. Available from: https://data.worldbank.org/indicator/EN.POP.SLUM.UR.ZS?end=2020&locations=8S&most_recent_value_desc=true&start=2020&view=map&year=2020

18. de Snyder VNS, Friel S, Fotso JC, Khadr Z, Meresman S, Monge P, et al. Social Conditions and Urban Health Inequities: Realities, Challenges and Opportunities to Transform the Urban Landscape through Research and Action. J Urban Health. 2011 Dec 1;88(6):1183–93.

19. Mehrolhasani MH, Yazdi-Feyzabadi V, Ghasemi S. Community empowerment for health promotion in slums areas: A narrative review with emphasis on challenges and interventions. J Educ Health Promot. 2021 Jul 30;10:263.

20. Sheuya SA. Improving the Health and Lives of People Living in Slums. Ann N Y Acad Sci. 2008;1136(1):298–306.

21. Ahmed S, Meenar M. Just Sustainability in the Global South: A Case Study of the Megacity of Dhaka. J Dev Soc. 2018 Dec 1;34(4):401–24.

22. Adams AM, Rashid SF. Mobilising demand for primary health care services among urban slums: insights from a case study in Bangladesh. Dev Pract. 2021 Aug 18;31(6):726–38.

23. Vearey J, Luginaah I, Magitta NF, Shilla DJ, Oni T. Urban health in Africa: a critical global public health priority. BMC Public Health. 2019 Mar 25;19(1):340.

24. Grundy J, Wang X, Hirabayashi KC, Duncan R, Bersonda D, Eltayeb AO, et al. Health and immunisation services for the urban poor in selected countries of Asia. Infect Dis Poverty. 2019 Apr 18;8(1):26.

25. Otmani del Barrio M, Simard F, Caprara A. Supporting and strengthening research on urban health interventions for the prevention and control of vector-borne and other infectious diseases of poverty: scoping reviews and research gap analysis. Infect Dis Poverty. 2018 Sep 3;7(1):94.

26. Zinszer K, Caprara A, Lima A, Degroote S, Zahreddine M, Abreu K, et al. Sustainable, healthy cities: protocol of a mixed methods evaluation of a cluster randomized controlled trial for Aedes control in Brazil using a community mobilization approach. Trials. 2020 Feb 14;21(1):182.

27. Dion A, Carini-Gutierrez A, Jimenez V, Ben Ameur A, Robert E, Joseph L, et al. Weight of Evidence: Participatory Methods and Bayesian Updating to Contextualize Evidence Synthesis in Stakeholders' Knowledge. J Mix Methods Res [Internet]. 2021 Oct 13 [cited 2021 Nov 1]; Available from: https://doi.org/10.1177/15586898211037412

28. Sarmiento I, Paredes-Solís S, Dion A, Silver H, Vargas E, Cruz P, et al. Maternal health and Indigenous traditional midwives in southern Mexico: contextualisation of a scoping review. BMJ Open. 2021 Dec;11(12):e054542.

29. Peters M, Godfrey C, McInerney P, Munn Z, Trico A, Khalil H. Chapter 11: Scoping Reviews. In: Aromataris E, Munn Z, editors. JBI Manual for Evidence Synthesis [Internet]. JBI; 2020 [cited 2022 Feb 5]. Available from: https://wiki.jbi.global/display/MANUAL/Chapter+11%3A+Scoping+reviews

30. Peters MDJ, Godfrey C, McInerney P, Khalil H, Larsen P, Marnie C, et al. Best practice guidance and reporting items for the development of scoping review protocols. JBI Evid Synth. 2022 Apr;20(4):953–68.

31. Shamseer L, Moher D, Clarke M, Ghersi D, Liberati A, Petticrew M, et al. Preferred reporting items for systematic review and meta-analysis protocols (PRISMA-P) 2015:

For peer review only - http://bmjopen.bmj.com/site/about/guidelines.xhtml

elaboration and explanation. BMJ. 2015 Jan 2;349:g7647.
32. Tricco AC, Lillie E, Zarin W, O'Brien KK, Colquhoun H, Levac D, et al. PRISMA Extension for Scoping Reviews (PRISMA-ScR): Checklist and Explanation. Ann Intern Med. 2018 Oct 2;169(7):467–73.
33. Goodman RA, Bunnell R, Posner SF. What is "community health"? Examining the meaning of an evolving field in public health. Prev Med. 2014 Oct 1;67:S58–61.
34. MacQueen K, McLellan E, Metzger DS, Kegeles S, Strauss RP, Scotti R, et al. What Is Community? An Evidence-Based Definition for Participatory Public Health. Am J Public Health. 2001;91(12):1929–38.
35. World Health Organization. The 7th Global Conference on Health Promotion - Community Empowerment [Internet]. World Health Organization. 2009 [cited 2022 Aug 5]. Available from: https://www.who.int/teams/health-promotion/enhanced-wellbeing/seventh-global-conference/community-empowerment
36. The World Bank. World Bank Country and Lending Groups [Internet]. The World Bank: Working for a World Free from Poverty. 2022 [cited 2022 Aug 25]. Available from: https://datahelpdesk.worldbank.org/knowledgebase/articles/906519-world-bank-country-and-lending-groups
37. Deuskar C. What does "urban" mean? [Internet]. World Bank Blogs. 2015 [cited 2022 Aug 25]. Available from: https://blogs.worldbank.org/sustainablecities/what-does-urban-mean
38. Brunton G, Thomas J, O'Mara-Eves A, Jamal F, Oliver S, Kavanagh J. Narratives of community engagement: a systematic review-derived conceptual framework for public health interventions. [Review]. BMC Public Health. 2017 Dec;17(1):944.
39. Draper AK, Hewitt G, Rifkin S. Chasing the dragon: Developing indicators for the assessment of community participation in health programmes. Soc Sci Med. 2010 Sep;71(6):1102–9.
40. Key KD, Furr-Holden D, Lewis EY, Cunningham R, Zimmerman MA, Johnson-Lawrence V, et al. The Continuum of Community Engagement in Research: A Roadmap for Understanding and Assessing Progress. Prog Community Health Partnersh Res Educ Action. 2019;13(4):427–34.
41. Wallerstein N, Duran B. The theoretical, historical, and practice roots of CBPR. In: Community-Based Participatory Research for Health: Advancing Social and Health Equity. Third Edition. San Francisco (US): Jossey-Bass: A Wiley Brand; 2008. p. 17–30.
42. The Cochrane Collaboration. LMIC Filters [Internet]. Cochrane Effective Practice and Organisation of Care: Trusted evidence, informed decisions, better health. 2022 [cited 2022 Aug 25]. Available from: https://epoc.cochrane.org/lmic-filters
43. Morris M, Boruff JT, Gore GC. Scoping reviews: establishing the role of the librarian. J Med Libr Assoc. 2016;104(4):346–53.
44. Veritas Health Innovation. Covidence systematic review software [Internet]. Melbourne (Australia); 2022. Available from: www.covidence.org
45. Page A. Unnatural and Unequal: Social Determinants of Gender Inequality and Health and Their Impact on Disaster Management Interventions in Bangladesh [Internet] [Thesis]. Université d'Ottawa / University of Ottawa; 2015 [cited 2021 Jun 15]. Available from: http://ruor.uottawa.ca/handle/10393/33186
46. Sarmiento I, Cockcroft A, Dion A, Paredes-Solís S, De Jesús-García A, Melendez D, et al. Combining conceptual frameworks on maternal health in Indigenous communities - Fuzzy cognitive mapping using participant and operator-independent weighting. Field

For peer review only

For peer review only - http://bmjopen.bmj.com/site/about/guidelines.xhtml

Methods. 2022;34(3):223–39.
47. Andersson N, Silver H. Fuzzy cognitive mapping: An old tool with new uses in nursing research. J Adv Nurs. 2019 Dec;75(12):3823–30.
48. Gray S, Gray S, De Kok JL, Helfgott A, O'Dwyer B, Jordan R, et al. Using fuzzy cognitive mapping as a participatory approach to analyze change, preferred states, and perceived resilience of social-ecological systems. Ecol Soc [Internet]. 2015 [cited 2021 Nov 4];20(2). Available from: https://www.ecologyandsociety.org/vol20/iss2/art11/
49. Sarmiento I, Paredes-Solis S, Morris M, Pimentel J, Cockcroft A, Andersson N. Factors influencing maternal health in indigenous communities with presence of traditional midwifery in the Americas: protocol for a scoping review. BMJ Open. 2020;10(10):e037922.
50. Kosko B. Fuzzy cognitive maps. Int J Man-Mach Stud. 1986 Jan 1;24(1):65–75.
51. Kosko B. Hidden patterns in combined and adaptive knowledge networks. Int J Approx Reason. 1988 Oct 1;2(4):377–93.
52. Andersson N, Mitchell S. Epidemiological geomatics in evaluation of mine risk education in Afghanistan: introducing population weighted raster maps. Int J Health Geogr. 2006 Jan 3;5(1):1.
53. Sarmiento I, Paredes-Solis S, Loutfi D, Dion A, Cockcroft A, Andersson N. Fuzzy cognitive mapping and soft models of indigenous knowledge on maternal health in Guerrero, Mexico. BMC Med Res Methodol. 2020;20(1):125.
54. Niesink P, Poulin K, Šajna M. Computing transitive closure of bipolar weighted digraphs. Discrete Appl Math. 2013 Jan 1;161(1):217–43.
55. Harris ZS. Discourse Analysis. Language. 1952;28(1):1–30.
56. Arksey H, O'Malley L. Scoping studies: towards a methodological framework. Int J Soc Res Methodol. 2005 Feb 1;8(1):19–32.
57. Boyko JA, Lavis JN, Abelson J, Dobbins M, Carter N. Deliberative dialogues as a mechanism for knowledge translation and exchange in health systems decision-making. Soc Sci Med. 2012;75(11):1938–45.
58. Dion A, Nakajima A, McGee A, Andersson N. How Adolescent Mothers Interpret and Prioritize Evidence About Perinatal Child Protection Involvement: Participatory Contextualization of Published Evidence. Child Adolesc Soc Work J [Internet]. 2022 Jul 13 [cited 2022 Sep 21]; Available from: https://link.springer.com/10.1007/s10560-022-00865-1

For peer review only

For peer review only - http://bmjopen.bmj.com/site/about/guidelines.xhtml

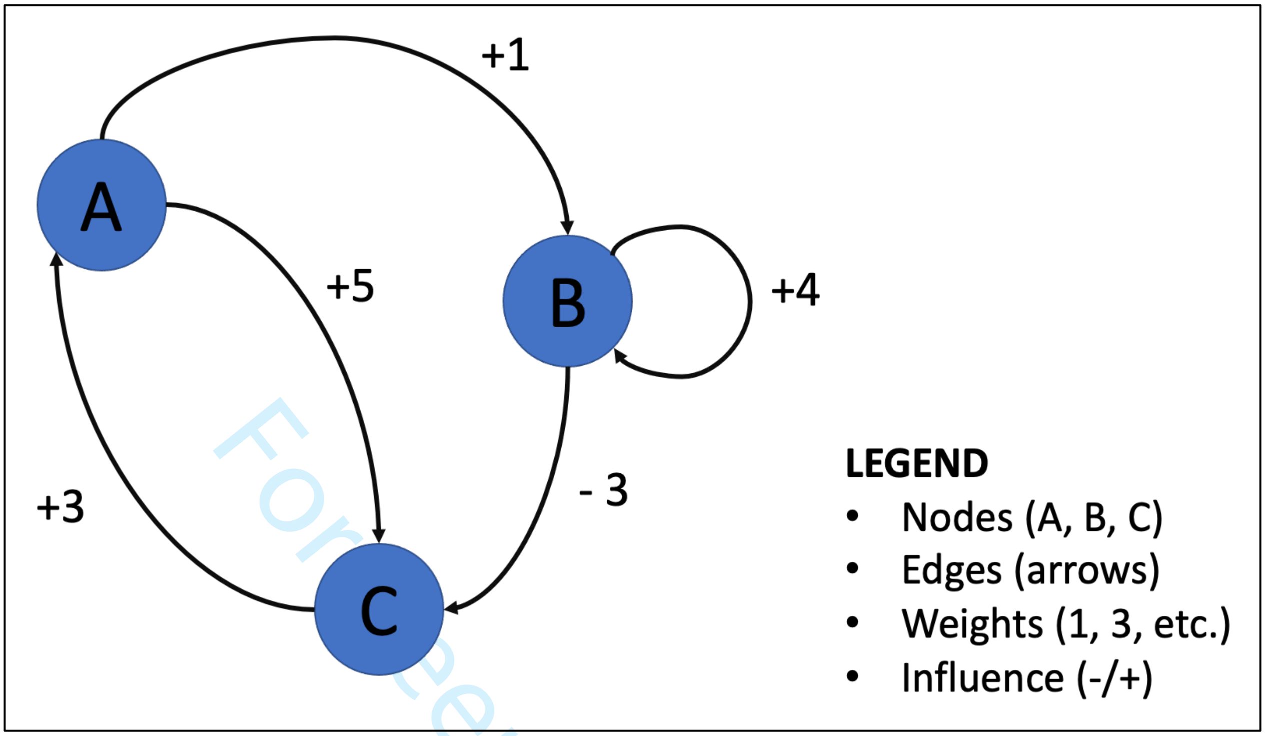

For peer review only

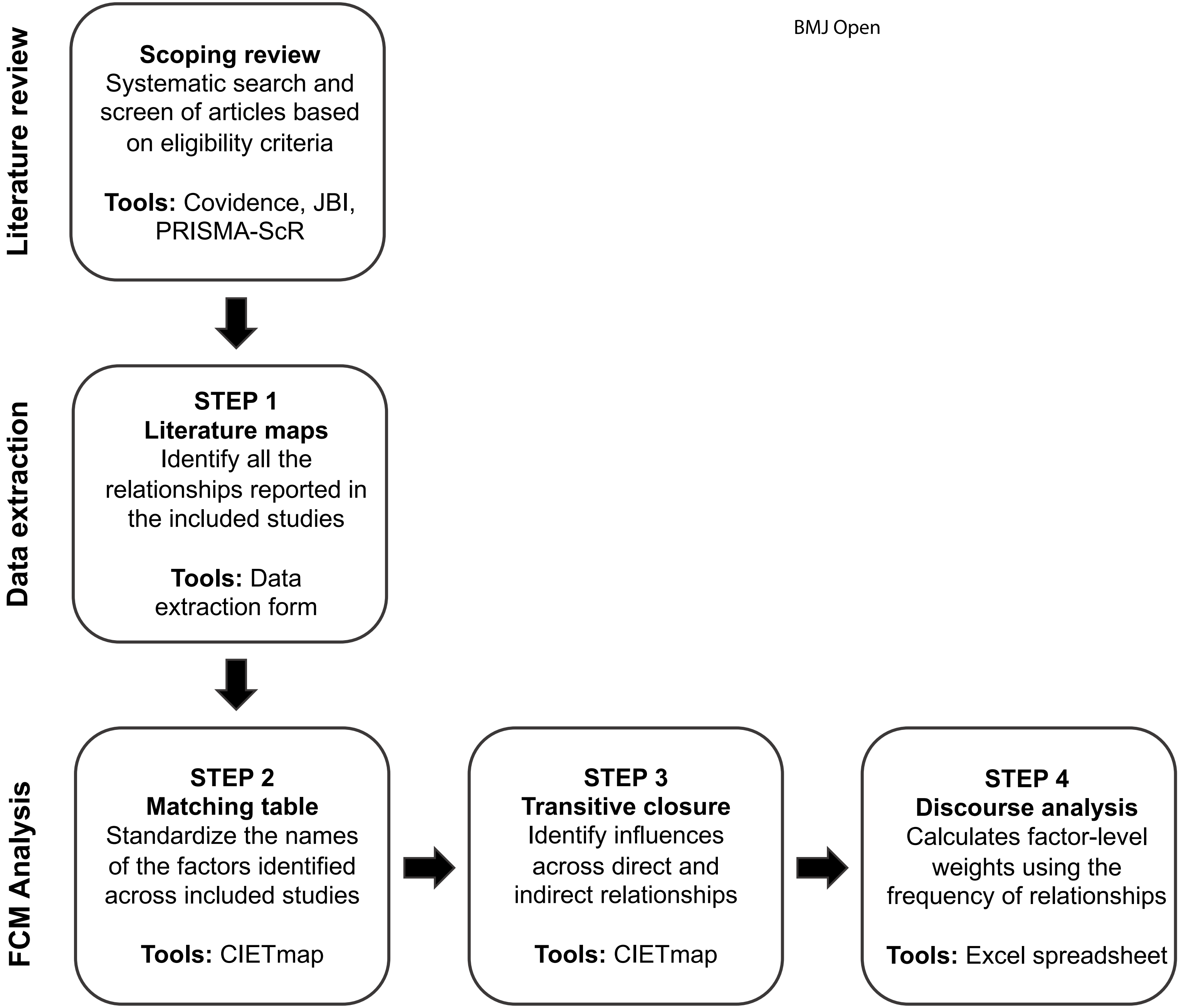

For peer review only - http://bmjopen.bmj.com/site/about/guidelines.xhtml

Figure 2. Steps of the fuzzy cognitive mapping process for the scoping review.

PRISMA-P 2015 Checklist

This checklist has been adapted for use with protocol submissions to *Systematic Reviews* from Table 3 in Moher D et al: Preferred reporting items for systematic review and meta-analysis protocols (PRISMA-P) 2015 statement. *Systematic Reviews* 2015 **4**:1

Section/topic	#	Checklist item	Information reported: Yes	Information reported: No	Line number(s)
**ADMINISTRATIVE INFORMATION**					
**Title**					
Identification	1a	Identify the report as a protocol of a systematic review	☒	☐	Page 1
Update	1b	If the protocol is for an update of a previous systematic review, identify as such	☐	☒	N/A
**Registration**	2	If registered, provide the name of the registry (e.g., PROSPERO) and registration number in the Abstract	☐	☒	N/A
**Authors**					
Contact	3a	Provide name, institutional affiliation, and e-mail address of all protocol authors; provide physical mailing address of corresponding author	☒	☐	Page 1
Contributions	3b	Describe contributions of protocol authors and identify the guarantor of the review	☒	☐	Page 12
**Amendments**	4	If the protocol represents an amendment of a previously completed or published protocol, identify as such and list changes; otherwise, state plan for documenting important protocol amendments	☐	☒	N/A
**Support**					
Sources	5a	Indicate sources of financial or other support for the review	☒	☐	Page 12
Sponsor	5b	Provide name for the review funder and/or sponsor	☒	☐	Page 12
Role of sponsor/funder	5c	Describe roles of funder(s), sponsor(s), and/or institution(s), if any, in developing the protocol	☒	☐	Page 12
**INTRODUCTION**					
**Rationale**	6	Describe the rationale for the review in the context of what is already known	☒	☐	Page 3 (bottom)
**Objectives**	7	Provide an explicit statement of the question(s) the review will address with reference to participants, interventions, comparators, and outcomes (PICO)	☒	☐	Page 4 (bottom)
**METHODS**					

For peer review only

BioMed Central The Open Access Publisher

Section/topic	#	Checklist item	Information reported Yes	Information reported No	Line number(s)
**Eligibility criteria**	8	Specify the study characteristics (e.g., PICO, study design, setting, time frame) and report characteristics (e.g., years considered, language, publication status) to be used as criteria for eligibility for the review	☒	☐	Pages 5-6
**Information sources**	9	Describe all intended information sources (e.g., electronic databases, contact with study authors, trial registers, or other grey literature sources) with planned dates of coverage	☒	☐	Page 5 (top); Page 6 (bottom)
**Search strategy**	10	Present draft of search strategy to be used for at least one electronic database, including planned limits, such that it could be repeated	☒	☐	Pages 6-8
STUDY RECORDS					
Data management	11a	Describe the mechanism(s) that will be used to manage records and data throughout the review	☒	☐	Page 8
Selection process	11b	State the process that will be used for selecting studies (e.g., two independent reviewers) through each phase of the review (i.e., screening, eligibility, and inclusion in meta-analysis)	☒	☐	Page 8
Data collection process	11c	Describe planned method of extracting data from reports (e.g., piloting forms, done independently, in duplicate), any processes for obtaining and confirming data from investigators	☒	☐	Pages 8-9
**Data items**	12	List and define all variables for which data will be sought (e.g., PICO items, funding sources), any pre-planned data assumptions and simplifications	☒	☐	Page 9 (point e)
**Outcomes and prioritization**	13	List and define all outcomes for which data will be sought, including prioritization of main and additional outcomes, with rationale	☒	☐	Pages 8-9 (points of data extraction form)
**Risk of bias in individual studies**	14	Describe anticipated methods for assessing risk of bias of individual studies, including whether this will be done at the outcome or study level, or both; state how this information will be used in data synthesis	☐	☒	N/A
DATA					
**Synthesis**	15a	Describe criteria under which study data will be quantitatively synthesized	☐	☒	N/A
	15b	If data are appropriate for quantitative synthesis, describe planned summary measures, methods of handling data, and methods of combining data from studies, including any planned exploration of consistency (e.g., I^2, Kendall's tau)	☐	☒	N/A
	15c	Describe any proposed additional analyses (e.g., sensitivity or subgroup analyses, meta-regression)	☐	☒	N/A
	15d	If quantitative synthesis is not appropriate, describe the type of summary planned	☒	☐	Pages 9-10
**Meta-bias(es)**	16	Specify any planned assessment of meta-bias(es) (e.g., publication bias across studies, selective reporting within studies)	☐	☒	N/A
**Confidence in cumulative evidence**	17	Describe how the strength of the body of evidence will be assessed (e.g., GRADE)	☐	☒	N/A

For peer review only

BioMed Central
The Open Access Publisher

For peer review only

For peer review only - http://bmjopen.bmj.com/site/about/guidelines.xhtml

BioMed Central
The Open Access Publisher
